# Ranking non-synonymous single nucleotide polymorphisms based on disease concepts

**DOI:** 10.1186/1479-7364-8-11

**Published:** 2014-06-30

**Authors:** Hashem A Shihab, Julian Gough, Matthew Mort, David N Cooper, Ian NM Day, Tom R Gaunt

**Affiliations:** 1 Bristol Centre for Systems Biomedicine and MRC Integrative Epidemiology Unit, School of Social and Community Medicine, University of Bristol, Oakfield House, Oakfield Grove, Bristol BS8 2BN, UK; 2 Department of Computer Science, University of Bristol, The Merchant Venturers Building, Bristol BS8 1UB, UK; 3 Institute of Medical Genetics, School of Medicine, Cardiff University, Cardiff CF14 4XN, UK

**Keywords:** SNV, nsSNPs, Disease-causing, Disease-specific, FATHMM, HMMs, SIFT, PolyPhen, Bioinformatics

## Abstract

As the number of non-synonymous single nucleotide polymorphisms (nsSNPs) identified through whole-exome/whole-genome sequencing programs increases, researchers and clinicians are becoming increasingly reliant upon computational prediction algorithms designed to prioritize potential functional variants for further study. A large proportion of existing prediction algorithms are ‘disease agnostic’ but are nevertheless quite capable of predicting when a mutation is likely to be deleterious. However, most clinical and research applications of these algorithms relate to specific diseases and would therefore benefit from an approach that discriminates between functional variants specifically related to that disease from those which are not. In a whole-exome/whole-genome sequencing context, such an approach could substantially reduce the number of false positive candidate mutations. Here, we test this postulate by incorporating a disease-specific weighting scheme into the Functional Analysis through Hidden Markov Models (FATHMM) algorithm. When compared to traditional prediction algorithms, we observed an overall reduction in the number of false positives identified using a disease-specific approach to functional prediction across 17 distinct disease concepts/categories. Our results illustrate the potential benefits of making disease-specific predictions when prioritizing candidate variants in relation to specific diseases. A web-based implementation of our algorithm is available at http://fathmm.biocompute.org.uk.

## Background

The average human exome harbours around 20,000 single nucleotide variants (SNVs), of which approximately half are annotated as non-synonymous single nucleotide polymorphisms (nsSNPs) [1]. However, characterizing the functional consequences of nsSNPs by direct laboratory experimentation is both time consuming and expensive. Therefore, computational prediction algorithms capable of predicting and/or prioritizing putatively functional variants for further experimentation are becoming increasingly important.

There is a plethora of computational prediction algorithms capable of analysing the functional consequences of nsSNPs [2]. One of these methods is the Functional Analysis through Hidden Markov Models (FATHMM) algorithm [3]: a sequence-based method which combines evolutionary conservation in homologous (both orthologous and/or paralogous) sequences with ‘pathogenicity weights’, representing the overall tolerance of proteins (and their component domains) to mutations. Using our original weighting scheme (adjusted for inherited disease mutations), we observed an improved predictive performance over alternative computational prediction algorithms using a ‘gold-standard’ benchmark [4]. Nonetheless, these algorithms, including our own, were not designed to discriminate between nsSNPs influencing a specific disease (disease-specific) and other putative disease-causing/functional mutations (non-specific). For example, when tasked with discriminating between cancer-associated and other germline polymorphisms, these algorithms are capable of identifying a high proportion of cancer-promoting mutations. However, a large proportion of putative disease-causing (non-neoplastic) mutations are misclassified as having a role in carcinogenesis [5]. In both a clinical and a research context, these tools are commonly used to investigate the aetiology of specific diseases. We therefore believe that there is a significant need for disease-specific functional variant predictions.

To the best of our knowledge, computational prediction algorithms have been explored exclusively in a *gene-specific* manner, e.g. predicting the effects of nsSNPs in mismatch repair proteins [6,7]. The sole context in which *disease-specific* predictions have been developed is in the prediction of cancer-associated mutations [8-11]. In our previous work, we adapted our original algorithm by means of a cancer-specific weighting scheme and observed improved predictive performances over alternative (cancer-specific) computational prediction algorithms when predicting the functional consequences of cancer-associated nsSNPs [12]. We have now extended this concept to a novel and more comprehensive ‘disease-specific’ weighting scheme to investigate whether such an approach is capable of prioritizing nsSNPs based on 17 disease concepts/categories.

## Results

**Figure 1:**
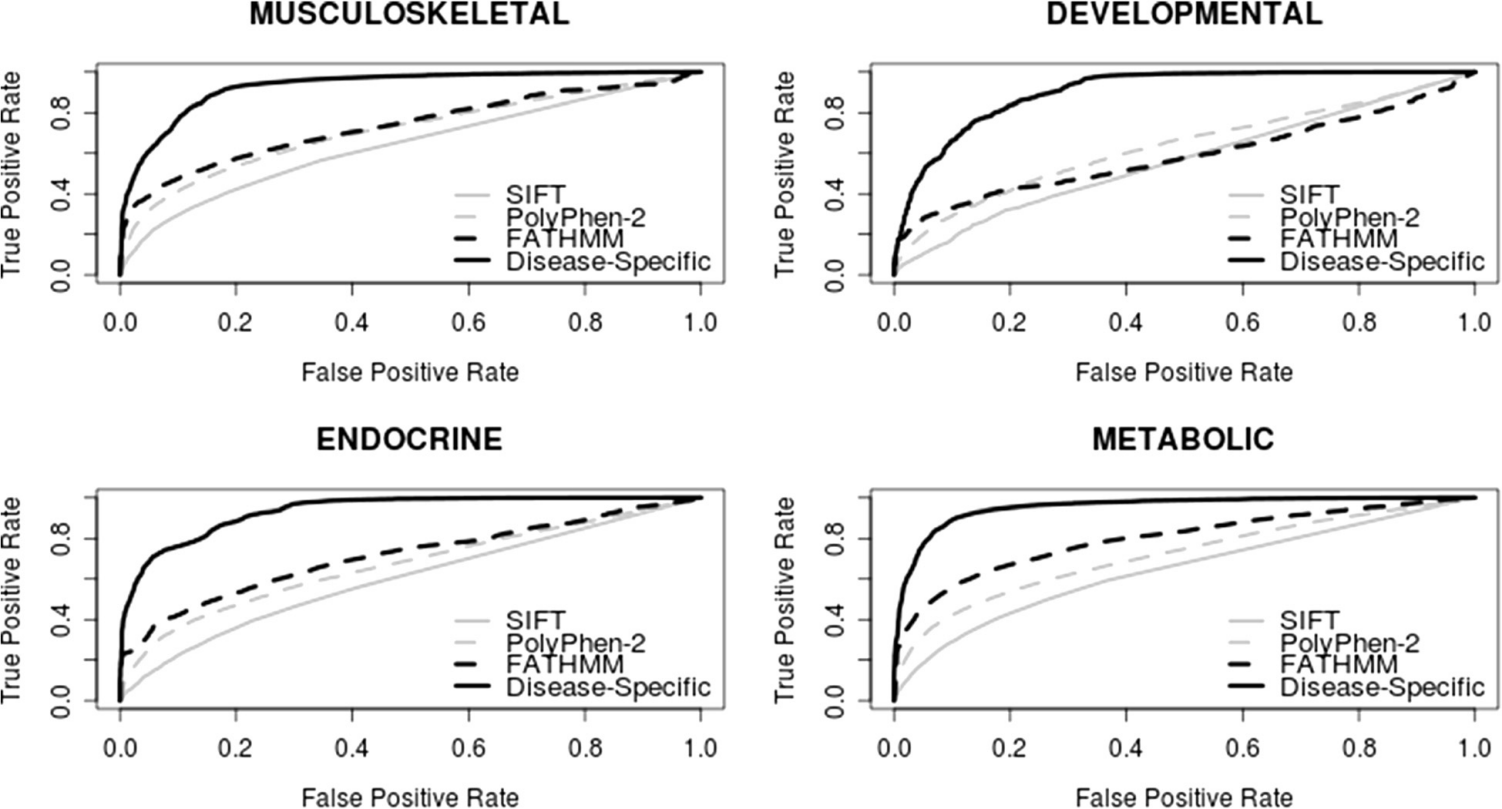
**Performance of disease-specific and generic computational prediction algorithms.** ROC curves for computational prediction algorithms when tasked with discriminating between disease-specific mutations and other germline variants (i.e. other disease-causing/neutral mutations).

In order to assess the potential benefits of making disease-specific predictions, we compared the performance of our disease-specific weighting scheme with the performance of our original algorithm (weighted for inherited disease mutations) and two (generic) computational prediction algorithms: SIFT [13] and PolyPhen-2 [14]. In our analysis, all generic prediction algorithms were found to be capable of discriminating between disease-causing mutations (i.e. both disease-specific and non-specific disease-causing mutations) and putative neutral polymorphisms (see Additional file 1: Supp. Info 1). However, our analysis showed that no distinction could be made between disease-specific and other non-specific disease-causing mutations when using these algorithms. For example, generic algorithms are incapable of discriminating between musculoskeletal-related variants and other disease-associated variants, thereby leading to high false positive rates (i.e. other disease-causing variants being incorrectly identified as being pathogenic with respect to musculoskeletal-related disease). On the other hand, it appears that a disease-specific approach to functional prediction is capable of distinguishing between disease-specific and other disease-causing mutations, thereby reducing the number of false positives identified and improving the overall performance of the algorithm. While our disease-specific approach is more specific than generic computational prediction algorithms, it would appear that this approach is also less sensitive. This general trend of greater specificity/less sensitivity was observed throughout the 17 disease concepts we tested (see Table 1 and Figure 1—data shown for musculoskeletal, developmental, endocrine and metabolic disorders; see Additional file 1: Supp. Info 2–18 for additional performance comparisons pertinent to the remaining disease concepts). These results illustrate the potential benefit of using a disease-specific approach to functional prediction when assessing nsSNPs in relation to specific diseases (by reducing the number of false positives identified); however, further work is needed to reduce the number of false negatives identified and improve sensitivity.

**Table 1:** Performance of computational prediction algorithms when discriminating between disease-specific variants and other disease-causing/neutral variants

**Algorithm**	**tp**	**fp**	**tn**	**fn**	**Accuracy**	**Precision**	**Specificity**	**Sensitivity**	**NPV**	**MCC**	**AUC**
Musculoskeletal											
SIFT	4,730	37,701	23,323	944	0.61	0.57	0.38	0.83	0.70	0.24	0.64
PolyPhen-2	5,278	44,047	34,859	714	0.66	0.61	0.44	0.88	0.79	0.36	0.71
FATHMM	5,902	51,596	29,202	201	0.66	0.60	0.36	*0.97*	*0.92*	0.41	0.73
Disease-Specific	4,120	3,123	77,675	1,983	*0.82*	*0.95*	*0.96*	0.68	0.75	*0.66*	*0.93*
Disease-Specific (20-fold)	-	-	-	-	0.80	0.92	0.94	0.66	0.74	0.63	-
Developmental											
SIFT	845	41,586	23,983	284	0.56	0.54	0.37	0.75	0.59	0.12	0.56
PolyPhen-2	920	48,405	35,337	236	0.61	0.58	0.42	0.80	0.67	0.23	0.63
FATHMM	1,006	52,429	33,278	188	0.62	0.58	0.39	*0.84*	*0.71*	0.26	0.59
Disease-Specific	621	710	84,997	573	*0.76*	*0.98*	*0.99*	0.52	0.67	*0.58*	*0.90*
Disease-Specific (20-fold)	-	-	-	-	0.74	0.97	0.99	0.49	0.66	0.55	-
Endocrine											
SIFT	3,084	39,347	23,443	824	0.58	0.56	0.37	0.79	0.64	0.18	0.60
PolyPhen-2	2,890	46,435	35,031	542	0.64	0.60	0.43	0.84	0.73	0.30	0.67
FATHMM	3,597	49,466	33,522	316	0.66	0.61	0.40	*0.92*	*0.83*	0.38	0.71
Disease-Specific	2,392	1,015	81,973	1,521	*0.80*	*0.98*	*0.99*	0.61	0.72	*0.65*	*0.94*
Disease-Specific (20-fold)	-	-	-	-	0.79	0.97	0.98	0.60	0.71	0.63	-
Metabolic											
SIFT	10,731	31,700	21,913	2,354	0.61	0.58	0.41	0.82	0.69	0.25	0.64
PolyPhen-2	11,337	37,988	33,788	1,785	0.67	0.62	0.47	0.86	0.78	0.36	0.72
FATHMM	13,068	39,914	33,271	648	0.70	0.64	0.45	*0.95*	*0.91*	0.47	0.80
Disease-Specific	10,767	3,209	69,976	2,949	*0.87*	*0.95*	*0.96*	0.78	0.82	*0.75*	*0.95*
Disease-Specific (20-fold)	-	-	-	-	0.86	0.94	0.95	0.77	0.81	0.74	-

In the above, tp, fp, tn and fn refer to the number of true positives, false positives, true negatives and false negatives observed, respectively. Accuracy, precision, specificity, sensitivity, negative predictive value (NPV) and Matthew's correlation coefficient (MCC) were calculated using normalized numbers. Italic font corresponds to the best performing method for a given statistic.

As our weighting scheme was derived using the same mutation data used to assess our method (albeit using a leave-one-out analysis), we recognize the potential for bias. Therefore, we also performed a 20-fold cross-validation analysis (see Table 1 and Additional file 1: Supp. Info 2–18). We observed no significant deviations in the performance measures reported and therefore concluded that the performance of our disease-specific approach is not an artefact of over-fitting. We also recognize that most of our algorithm's predictive power comes from our weighting scheme, i.e. it is the weighting scheme that allows us to differentiate between disease-associated variants and other disease-causing mutations. Therefore, we also compared our approach to a naive weighting scheme. Here, we used our weighting scheme (omitting sequence conservation) to derive a prediction score. Proteins, and their constituent domains, with a higher proportion of disease-associated mutations would predict all variants falling within them as *disease*, and those with a higher proportion of other disease-causing mutations/neutral polymorphisms would predict all variants as *neutral*. Overall, we observed a similar performance to that of our algorithm (see Additional file 1: Supp. Info 19–36). However, it should be noted that a naive approach is incapable of reliably discriminating between disease-associated mutations and other disease-causing variants as the weighting scheme becomes more balanced, whereas our disease-specific approach (which incorporates sequence conservation for prediction) appears to be less susceptible to balanced weights.

In order to facilitate the replication of our work, we have annotated SwissProt/TrEMBL disease variants (Release 2014_06) with the disease concepts used in our analysis and make this resource publically available at our website (http://fathmm.biocompute.org.uk). Using this dataset to train and test our algorithm, we observed similar performances to those reported above (see Additional file 2).

## Discussion

There is a plethora of computational prediction algorithms available to predict the functional consequences of nsSNPs [2]. However, these algorithms are not designed to distinguish between mutations related to a specific disease, or a group of related diseases (disease-specific), and other putative disease-causing (non-specific) mutations. As the cost of whole-exome/whole-genome sequencing falls, making these methods more amenable to use in a research or clinical context, the challenge of filtering true disease-causing candidate variants from other putative functional variants is likely to become increasingly important. In this work, we assessed the potential benefits of making disease-specific predictions (relevant to 17 disease categories) using the Functional Analysis through Hidden Markov Models (FATHMM) framework and observed an overall reduction in the number of false positives identified, thereby leading to improved specificity over traditional algorithms. However, we also observed an increase in the number of false negatives identified and conclude that additional work is needed to improve sensitivity and enhance the utility of our disease-specific approach. Nevertheless, there is potential to extend this approach to more specific categories for the purposes of enhancing clinical prediction.

An important consideration when evaluating the performance of computational prediction algorithms is the cross-validation dataset. Here, the performance of such algorithms should be trained and tested using different datasets (cross-validation). In order to alleviate the potential for bias in our results, we performed a 20-fold cross-validation procedure across our 17 disease concepts. From this analysis, we observed no significant deviations in the reported performance measures and therefore conclude that the performances observed were not an artefact of our disease-specific weighting scheme.

One of the major limitations of our disease-specific approach is that, in extreme cases, there is potential for dominating pathogenicity weights which could bias or exaggerate the effects of variants, e.g. when prioritizing variants in proteins and/or domains which have very strong associations with the disease concept under investigation. Here, the pathogenicity weights used could dominate the underlying amino acid probabilities (used to measure sequence conservation) and therefore bias the prediction. For example, when these weights are biased towards the disease concept, neutral polymorphisms falling within diverse regions of a protein/domain would be classified as ‘damaging’ as opposed to being classified as ‘benign’. As a consequence, our disease-specific models are best suited as a whole-genome/whole-exome prioritization method (hypothesis-free) and should be used with caution when prioritizing variants in a gene-specific manner. In an attempt to alleviate the potential effects of dominating pathogenicity weights, measures of sequence conservation are presented alongside our rankings so that spurious predictions can be assessed and ignored.

An alternative approach to our disease-specific weighting scheme is to filter putative disease-causing nsSNPs using the Gene Ontology [15]. However, this approach is dependent upon protein annotations being made available whereas our algorithm does not require prior information on protein function. Furthermore, users adopting this approach are required to select from a range of technical phrases, e.g. ‘negative regulation of cellular macromolecule biosynthetic process’ (GO: 2000113). In contrast, our disease-specific models do not require any formal knowledge on GO terms and biological processes, just an understanding of which model/concept best represents the disease under investigation. Our disease-specific models, including a high-throughput web-based implementation of our algorithm and a standalone software package, are available at http://fathmm.biocompute.org.uk.

## Methods

### Predicting the functional consequences of nsSNPs

The procedure for predicting the functional consequences of nsSNPs has been described in Shihab et al. [3]. In brief, an *ab initio* hidden Markov model (HMM), representing the multiple sequence alignment of homologous (both orthologous and/or paralogous) sequences within the SwissProt/TrEMBL [16] database, is constructed using the HMMER3 [17] software suite. In conjunction, protein domains from the SUPERFAMILY [18] and Pfam (Pfam-A and Pfam-B) [19] databases are annotated onto the full-length protein sequence. If the mutation falls within an annotated region, then the corresponding model is extracted and used alongside our *ab initio* model. Next, our algorithm combines sequence conservation, within the most informative model (as measured by the Kullback-Leibler divergence [20] from the SwissProt/TrEMBL amino acid composition), with pathogenicity weights, representing the overall tolerance of the corresponding model to mutations (Equation 1).

(1) $$\ln\frac{\left(1.0-P_{w}\right)\left(W_{n}+1.0\right)}{\left(1.0-P_{m}\right)\left(W_{d}+1.0\right)}$$

In Equation 1, *P*_w_ and *P*_m_ represent the probabilities for the wild-type and mutant amino acid residues, respectively, whereas *W*_d_ and *W*_n_ represent the relative frequencies of disease-associated and functionally neutral nsSNPs mapping onto the corresponding model, respectively. Here, we use inherited disease-causing nsSNPs annotated as DMs (damaging mutations) in the Human Gene Mutation Database (HGMD Pro 12.4 [21]) and putative neutral polymorphisms from the SwissProt/TrEMBL database [16] (Release 2013_04) to derive *W*_d_ and *W*_n_, respectively. The effect of our weighting scheme is as follows: when using pure conservation-based prediction methods, nsSNPs falling within diverse regions of the protein (or domain) are typically considered ‘neutral/benign’. However, our weighting scheme assesses the tolerance of the corresponding model (representing a protein or domain) to mutation and then adjusts a conservation-based prediction accordingly. For example, nsSNPs falling within *P53* (a well-established cancer gene) are penalized according to the gene's intolerance to mutation whereas nsSNPs falling within *MHC* (known to contain hypervariable regions) are not penalized given the gene's apparent tolerance to mutation.

### Incorporating a disease-specific weighting scheme

In order to derive a disease-specific weighting scheme, the phenotypes reported for inherited disease-causing nsSNPs listed as DMs (damaging mutations) in the Human Gene Mutation Database (HGMD Pro 12.4 [21]) were annotated using natural language processing against the Unified Medical Language System (UMLS [22]). These mutations were then grouped into 1 (or more) of 17 different root disease concepts, e.g. digestive disorders ([23] —see Table 2 for the complete list). For disease-specific predictions, our original weighting scheme (see Equation 1) is replaced with the relative frequencies of disease-specific mutations and other non-specific disease-causing mutations/neutral polymorphisms mapping onto the model, i.e. our pathogenic training set consists of disease-causing mutations related to the disease concept whereas our neutral training set comprises all other disease-causing mutations (not related to the corresponding disease concept) and putative neutral mutations. This disease-specific weighting scheme has the same effect as our original weighting scheme (i.e. to penalize specific variants); however, this approach penalizes just those variants falling within disease-specific susceptible proteins or domains and treats other disease-causing mutations as neutral polymorphisms (with respect to the disease concept under investigation).

**Table 2:** Summary of nsSNPs used in our disease-specific mutation datasets

**Dataset**	**Number of proteins**	**Number of amino acid substitutions**
Human Gene Mutation Database (HGMD)		
Blood	99	1,474
Blood coagulation	45	3,508
Developmental	188	1,199
Digestive	116	1,850
Ear, nose and throat	113	943
Endocrine	192	3,913
Eye	227	3,031
Genitourinary	166	3,031
Heart	247	3,743
Immune	75	1,293
Metabolic	485	13,797
Musculoskeletal	309	6,110
Nervous system	473	8,553
Psychiatric	163	747
Reproductive	88	883
Respiratory	44	775
Skin	164	3,183
SwissProt/TrEMBL		
Putative neutral polymorphisms	11,601	37,488

### Performance statistics

In accordance with published guidelines [24], the following six parameters are used to assess the performance of our disease-specific models:

$$\mathrm{Accuracy}=\frac{\mathrm{tp}+\mathrm{tn}}{\mathrm{tp}+\mathrm{tn}+\mathrm{fp}+\mathrm{fn}}$$

$$\mathrm{Precision}=\frac{\mathrm{tp}}{\mathrm{tp}+\mathrm{fp}}$$

$$\mathrm{Sensitivity}=\frac{\mathrm{tp}}{\mathrm{tp}+\mathrm{fn}}$$

$$\mathrm{Specificity}=\frac{\mathrm{tn}}{\mathrm{fp}+\mathrm{tn}}$$

$$\mathrm{NPV}=\frac{\mathrm{tn}}{\mathrm{tn}+\mathrm{fn}}$$

$$\mathrm{MCC}=\frac{\left(\mathrm{tp}\cdot \;\mathrm{tn}\right)-\left(\mathrm{fn}\;\cdot \mathrm{fp}\right)}{\sqrt{\left(\mathrm{tp}+\mathrm{fn}\right)\left(\mathrm{tp}+\mathrm{fp}\right)\left(\mathrm{tn}+\mathrm{fn}\right)\left(\mathrm{tn}+\mathrm{fp}\right)}}$$

In the aforementioned data, tp and fp refer to the number of true positives and false positives reported, respectively, whereas tn and fn refer to the number of true negatives and false negatives reported, respectively. Receiver operating characteristic (ROC) and area under the curve (AUC) analyses were performed using the ROCR software suite [25].

## Competing interests

The authors declare that they have no competing interests.

## Authors’ contributions

HAS participated in the design of the study and performed the analysis. TRG, JG and INMD participated in the design and coordination of the study. DNC and MM provided the training data and corresponding disease annotations. All authors read and approved the final manuscript.

## Supplementary Material

Additional file 1 **Performance of computational prediction algorithms.** This file reports the performance of computational prediction algorithms when tasked with discriminating between inherited disease-causing mutations, disease-specific mutations and neutral polymorphisms. Click here for file

Additional file 2 **Performance of computational prediction algorithms using SwissProt/TrEMBL.** This file reports the performance of our disease-specific algorithm and two generic computational prediction algorithms: SIFT and PolyPhen-2, when tasked with discriminating between disease-specific mutations and other disease-causing mutations/neutral polymorphisms in SwissProt/TrEMBL. Click here for file
