# Supplementary material for: Ranking non-synonymous single nucleotide polymorphisms based on disease concepts
Source: Hum Genomics. 2014 Jun 30;8(1):11. doi: 10.1186/1479-7364-8-11 (PMC4083756; doi:10.1186/1479-7364-8-11)
Supplement: Additional file 1 — Performance of computational prediction algorithms. This file reports the performance of computational prediction algorithms when tasked with discriminating between inherited disease-causing mutations, disease-specific mutations and neutral polymorphisms. [file 1479-7364-8-11-S1.docx]

Supp. Info 1. Performance of Computational Prediction Algorithms when tasked with discriminating between inherited disease-causing variants and putative neutral polymorphisms.


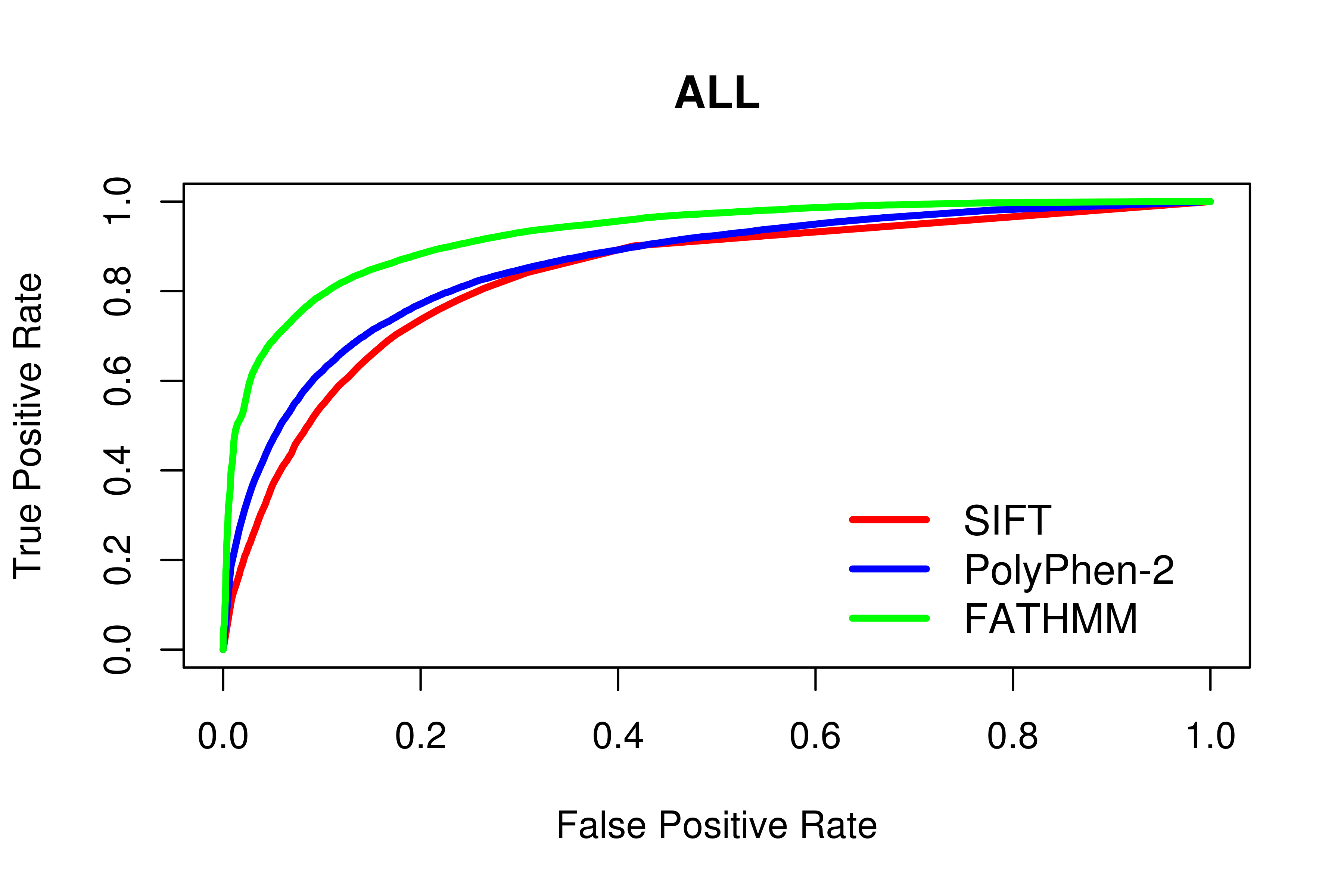


| Algorithm | tp | fp | tn | fn | Accuracy | Precision | Specificity | Sensitivity | NPV | MCC | AUC |
| --- | --- | --- | --- | --- | --- | --- | --- | --- | --- | --- | --- |
| SIFT | 38059 | 4372 | 13695 | 10572 | 0.77 | 0.76 | 0.76 | 0.78 | 0.78 | 0.54 | 0.83 |
| PolyPhen-2 | 39768 | 9557 | 27229 | 8344 | 0.78 | 0.76 | 0.74 | 0.83 | 0.81 | 0.57 | 0.86 |
| FATHMM | 44330 | 7007 | 29931 | 5633 | 0.85 | 0.82 | 0.81 | 0.89 | 0.88 | 0.7 | 0.93 |

In the above, *tp*, *fp*, *tn* and *fn* refer to the number of true positive, false positive, true negative and false negatives observed. *Accuracy*, *Precision*, *Specificity*, *Sensitivity*, Negative Predictive Value (*NPV*) and Matthew’s Correlation Coefficient (*MCC*) were calculated using normalized numbers.

Supp. Info 2. Performance of computational prediction algorithms when tasked with discriminating between “Blood” disease mutations and other disease-causing mutations/putative neutral polymorphisms.


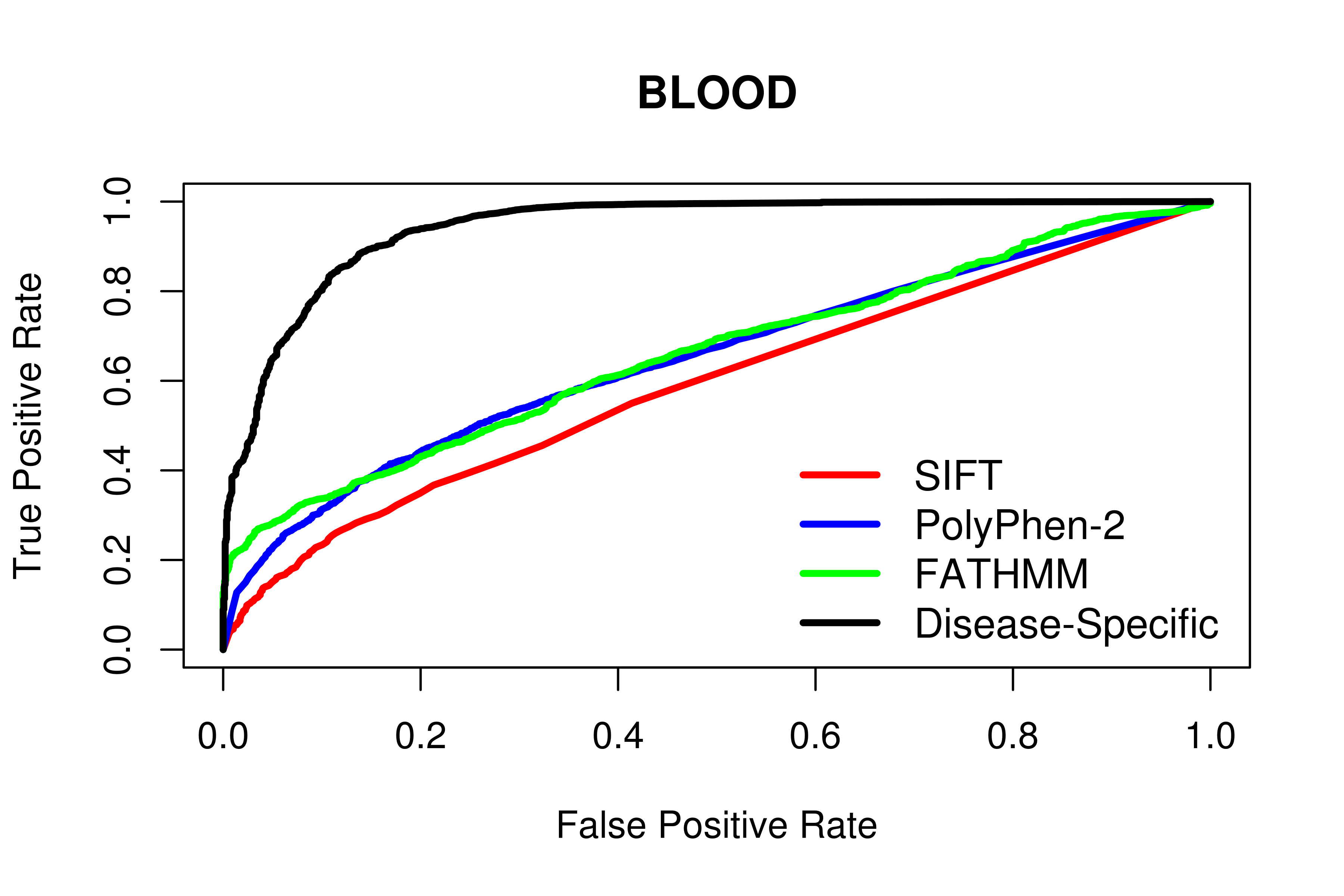


| Algorithm | tp | fp | tn | fn | Accuracy | Precision | Specificity | Sensitivity | NPV | MCC | AUC |
| --- | --- | --- | --- | --- | --- | --- | --- | --- | --- | --- | --- |
| SIFT | 1151 | 41280 | 23955 | 312 | 0.58 | 0.55 | 0.37 | 0.79 | 0.63 | 0.17 | 0.59 |
| PolyPhen-2 | 1191 | 48134 | 35309 | 264 | 0.62 | 0.59 | 0.42 | 0.82 | 0.7 | 0.26 | 0.65 |
| FATHMM | 1187 | 49345 | 36081 | 288 | 0.61 | 0.58 | 0.42 | 0.8 | 0.68 | 0.25 | 0.66 |
| Disease-Specific | 860 | 487 | 84939 | 615 | 0.79 | 0.99 | 0.99 | 0.58 | 0.7 | 0.63 | 0.94 |
| Disease-Specific (20-Fold) | - | - | - | - | 0.78 | 0.99 | 0.99 | 0.57 | 0.7 | 0.62 | - |

In the above, *tp*, *fp*, *tn* and *fn* refer to the number of true positive, false positive, true negative and false negatives observed. *Accuracy*, *Precision*, *Specificity*, *Sensitivity*, Negative Predictive Value (*NPV*) and Matthew’s Correlation Coefficient (*MCC*) were calculated using normalized numbers.

Supp. Info 3. Performance of computational prediction algorithms when tasked with discriminating between “Blood Coagulation” disease mutations and other disease-causing mutations/putative neutral polymorphisms.


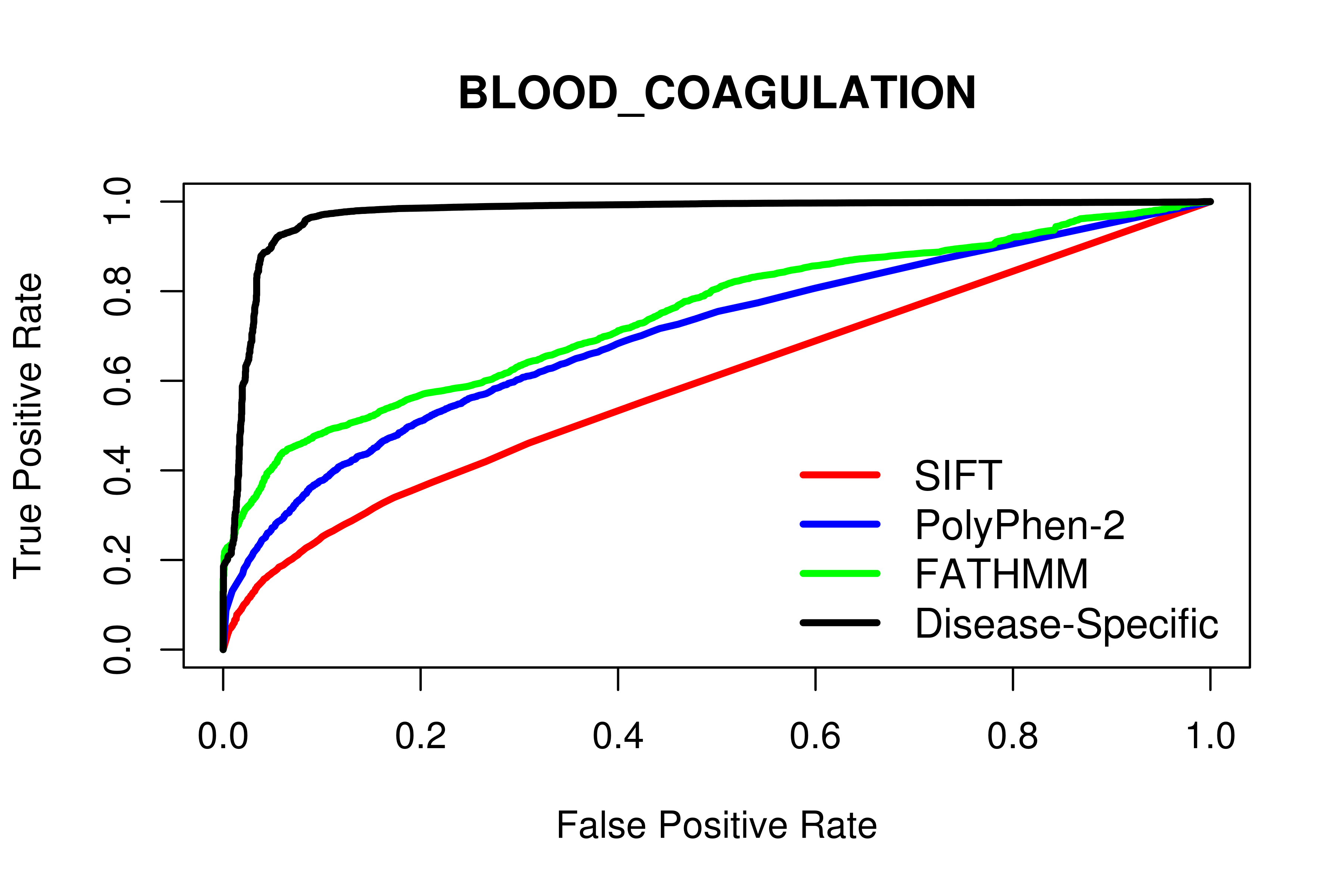


| Algorithm | tp | fp | tn | fn | Accuracy | Precision | Specificity | Sensitivity | NPV | MCC | AUC |
| --- | --- | --- | --- | --- | --- | --- | --- | --- | --- | --- | --- |
| SIFT | 2755 | 39676 | 23533 | 734 | 0.58 | 0.56 | 0.37 | 0.79 | 0.64 | 0.18 | 0.6 |
| PolyPhen-2 | 3019 | 46306 | 35094 | 479 | 0.65 | 0.6 | 0.43 | 0.86 | 0.76 | 0.33 | 0.7 |
| FATHMM | 3354 | 50843 | 32550 | 154 | 0.67 | 0.61 | 0.39 | 0.96 | 0.9 | 0.42 | 0.75 |
| Disease-Specific | 2885 | 1292 | 82101 | 623 | 0.9 | 0.98 | 0.98 | 0.82 | 0.85 | 0.82 | 0.97 |
| Disease-Specific (20-Fold) | - | - | - | - | 0.9 | 0.97 | 0.98 | 0.81 | 0.84 | 0.8 | - |

In the above, *tp*, *fp*, *tn* and *fn* refer to the number of true positive, false positive, true negative and false negatives observed. *Accuracy*, *Precision*, *Specificity*, *Sensitivity*, Negative Predictive Value (*NPV*) and Matthew’s Correlation Coefficient (*MCC*) were calculated using normalized numbers.

Supp. Info 4. Performance of computational prediction algorithms when tasked with discriminating between “Developmental” disease mutations and other disease-causing mutations/putative neutral polymorphisms.


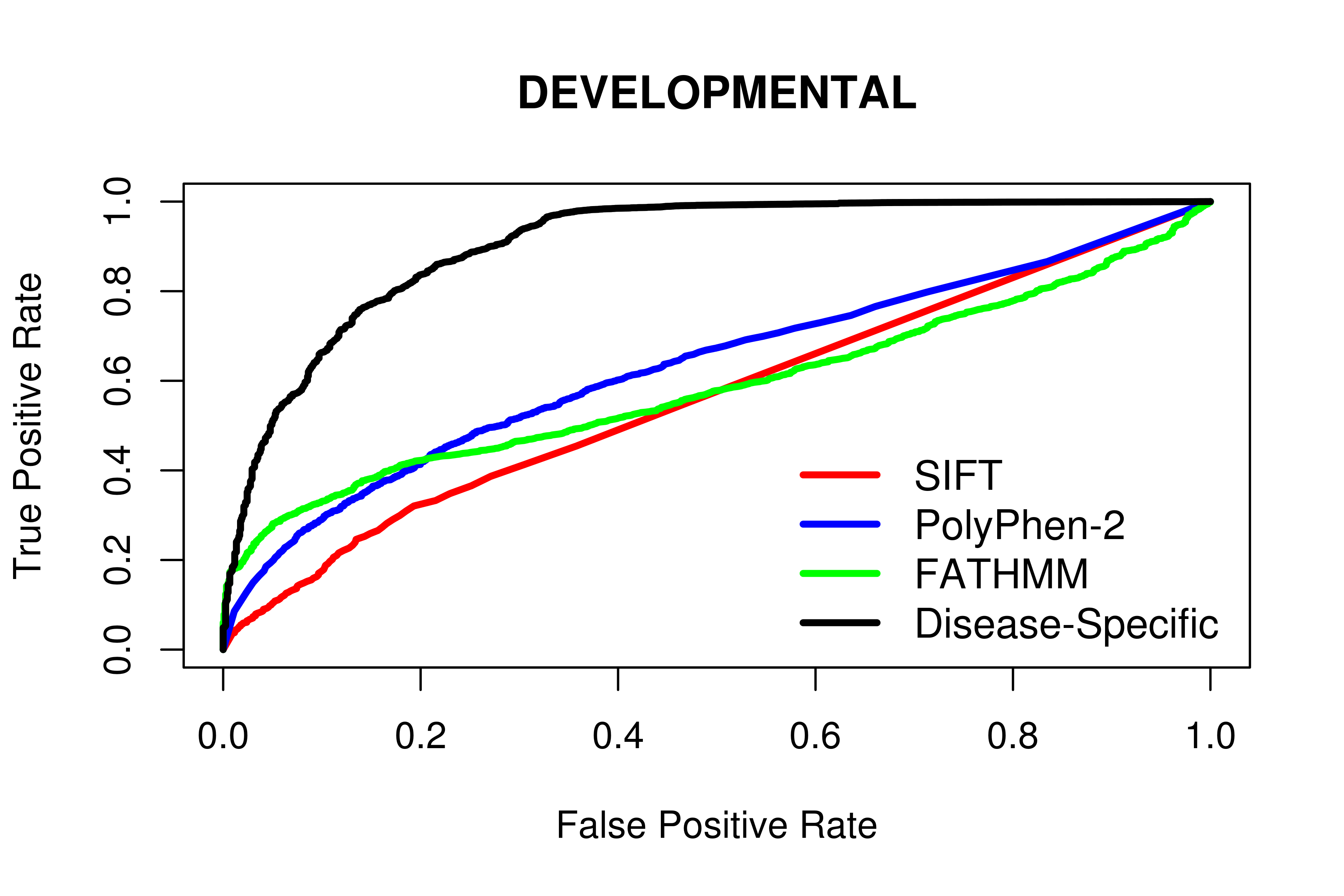


| Algorithm | tp | fp | tn | fn | Accuracy | Precision | Specificity | Sensitivity | NPV | MCC | AUC |
| --- | --- | --- | --- | --- | --- | --- | --- | --- | --- | --- | --- |
| SIFT | 845 | 41586 | 23983 | 284 | 0.56 | 0.54 | 0.37 | 0.75 | 0.59 | 0.12 | 0.56 |
| PolyPhen-2 | 920 | 48405 | 35337 | 236 | 0.61 | 0.58 | 0.42 | 0.8 | 0.67 | 0.23 | 0.63 |
| FATHMM | 1006 | 52429 | 33278 | 188 | 0.62 | 0.58 | 0.39 | 0.84 | 0.71 | 0.26 | 0.59 |
| Disease-Specific | 621 | 710 | 84997 | 573 | 0.76 | 0.98 | 0.99 | 0.52 | 0.67 | 0.58 | 0.9 |
| Disease-Specific (20-Fold) | - | - | - | - | 0.74 | 0.97 | 0.99 | 0.49 | 0.66 | 0.55 | - |

In the above, *tp*, *fp*, *tn* and *fn* refer to the number of true positive, false positive, true negative and false negatives observed. *Accuracy*, *Precision*, *Specificity*, *Sensitivity*, Negative Predictive Value (*NPV*) and Matthew’s Correlation Coefficient (*MCC*) were calculated using normalized numbers.

Supp. Info 5. Performance of computational prediction algorithms when tasked with discriminating between “Digestive” disease mutations and other disease-causing mutations/putative neutral polymorphisms.


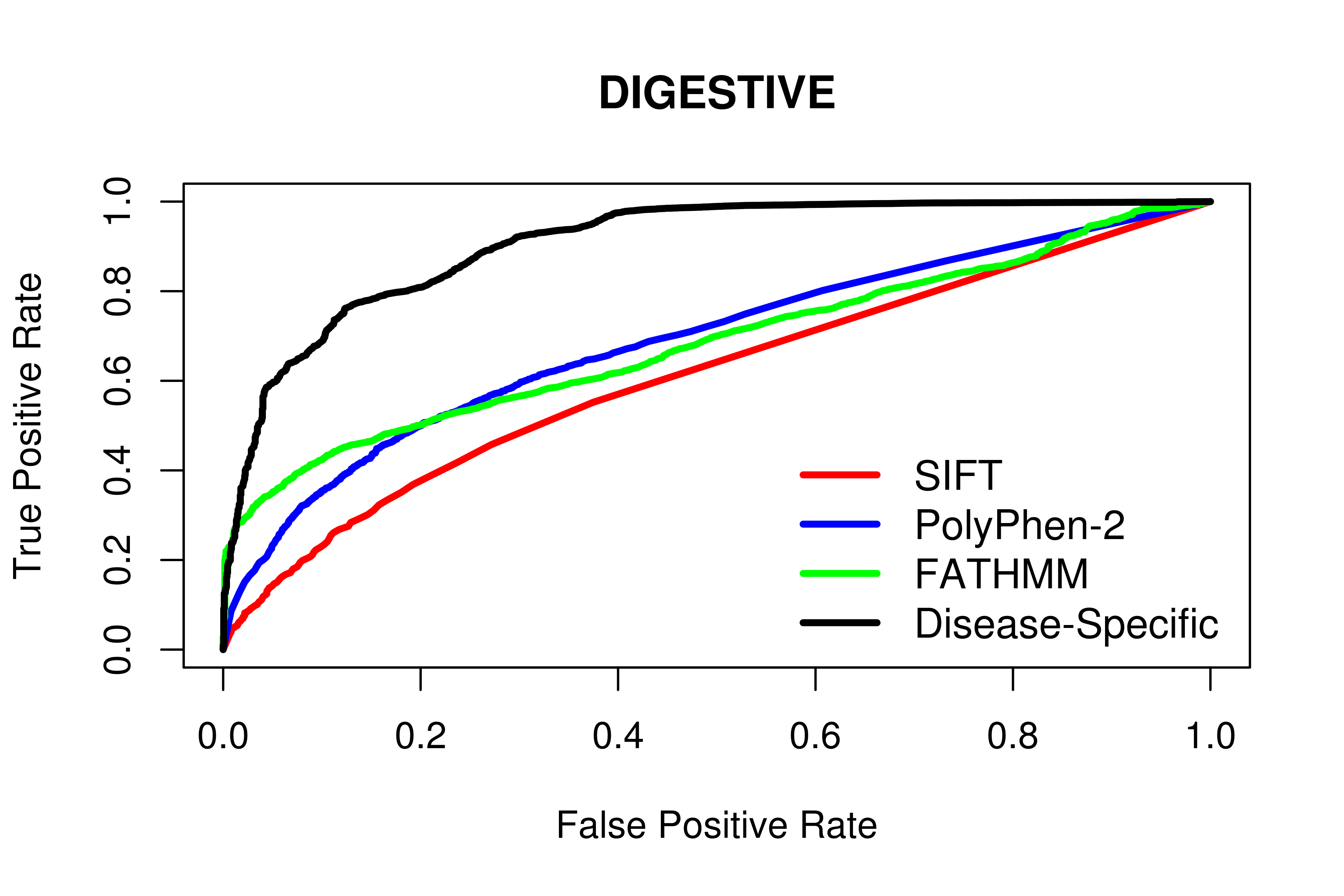


| Algorithm | tp | fp | tn | fn | Accuracy | Precision | Specificity | Sensitivity | NPV | MCC | AUC |
| --- | --- | --- | --- | --- | --- | --- | --- | --- | --- | --- | --- |
| SIFT | 1444 | 40987 | 23923 | 344 | 0.59 | 0.56 | 0.37 | 0.81 | 0.66 | 0.2 | 0.61 |
| PolyPhen-2 | 1496 | 47829 | 35319 | 254 | 0.64 | 0.6 | 0.42 | 0.85 | 0.75 | 0.31 | 0.69 |
| FATHMM | 1662 | 52450 | 32663 | 126 | 0.66 | 0.6 | 0.38 | 0.93 | 0.84 | 0.37 | 0.68 |
| Disease-Specific | 844 | 734 | 84379 | 944 | 0.73 | 0.98 | 0.99 | 0.47 | 0.65 | 0.54 | 0.91 |
| Disease-Specific (20-Fold) | - | - | - | - | 0.72 | 0.97 | 0.99 | 0.45 | 0.64 | 0.52 | - |

In the above, *tp*, *fp*, *tn* and *fn* refer to the number of true positive, false positive, true negative and false negatives observed. *Accuracy*, *Precision*, *Specificity*, *Sensitivity*, Negative Predictive Value (*NPV*) and Matthew’s Correlation Coefficient (*MCC*) were calculated using normalized numbers.

Supp. Info 6. Performance of computational prediction algorithms when tasked with discriminating between “Ear, Nose and Throat” disease mutations and other disease-causing mutations/putative neutral polymorphisms.


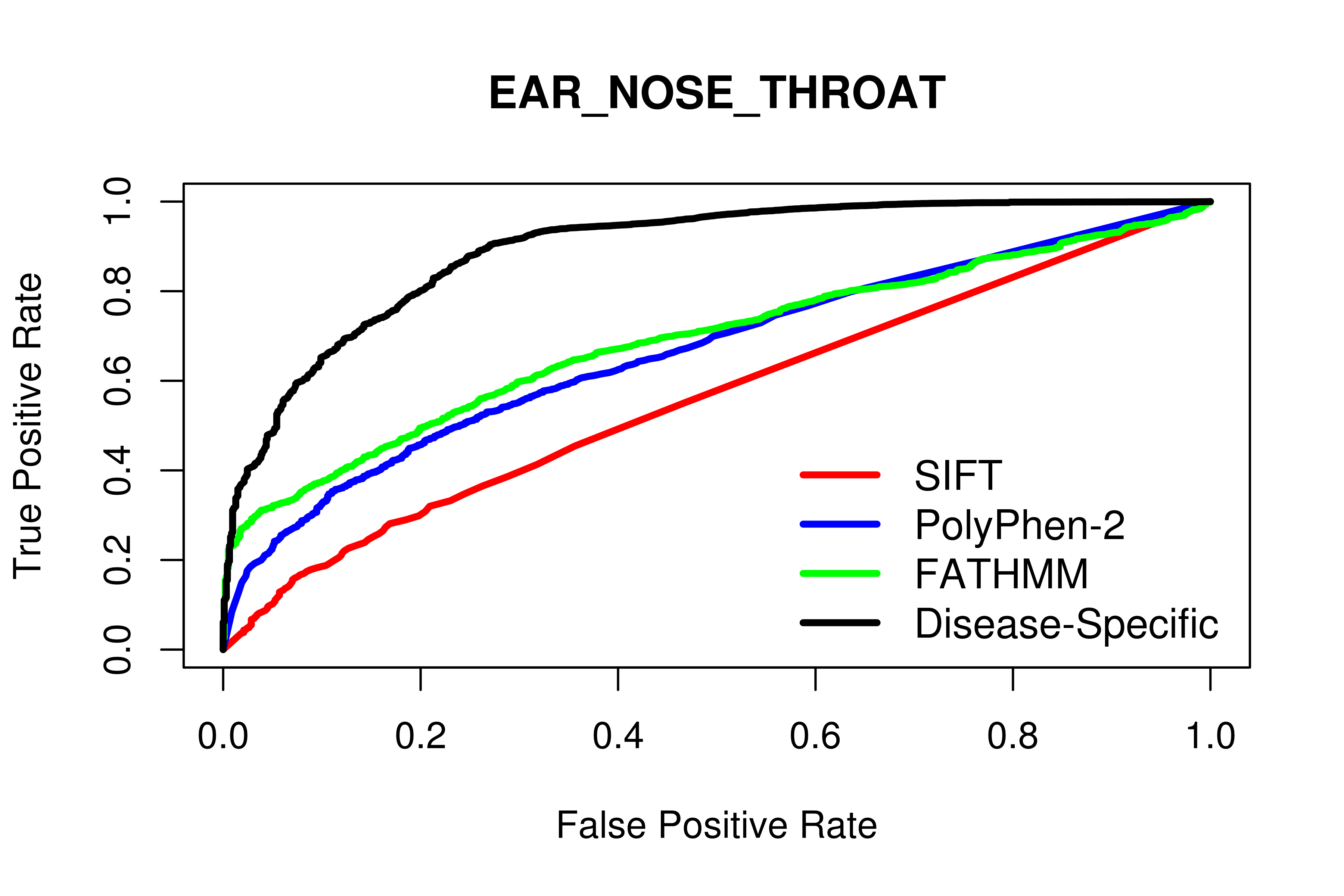


| Algorithm | tp | fp | tn | fn | Accuracy | Precision | Specificity | Sensitivity | NPV | MCC | AUC |
| --- | --- | --- | --- | --- | --- | --- | --- | --- | --- | --- | --- |
| SIFT | 673 | 41758 | 24027 | 240 | 0.55 | 0.54 | 0.37 | 0.74 | 0.58 | 0.11 | 0.56 |
| PolyPhen-2 | 751 | 48574 | 35417 | 156 | 0.62 | 0.59 | 0.42 | 0.83 | 0.71 | 0.27 | 0.67 |
| FATHMM | 681 | 36967 | 48994 | 259 | 0.65 | 0.63 | 0.57 | 0.72 | 0.67 | 0.3 | 0.69 |
| Disease-Specific | 192 | 199 | 85762 | 748 | 0.6 | 0.99 | 1 | 0.2 | 0.56 | 0.33 | 0.89 |
| Disease-Specific (20-Fold) | - | - | - | - | 0.59 | 0.98 | 1 | 0.18 | 0.55 | 0.31 | - |

In the above, *tp*, *fp*, *tn* and *fn* refer to the number of true positive, false positive, true negative and false negatives observed. *Accuracy*, *Precision*, *Specificity*, *Sensitivity*, Negative Predictive Value (*NPV*) and Matthew’s Correlation Coefficient (*MCC*) were calculated using normalized numbers.

Supp. Info 7. Performance of computational prediction algorithms when tasked with discriminating between “Endocrine” disease mutations and other disease-causing mutations/putative neutral polymorphisms.


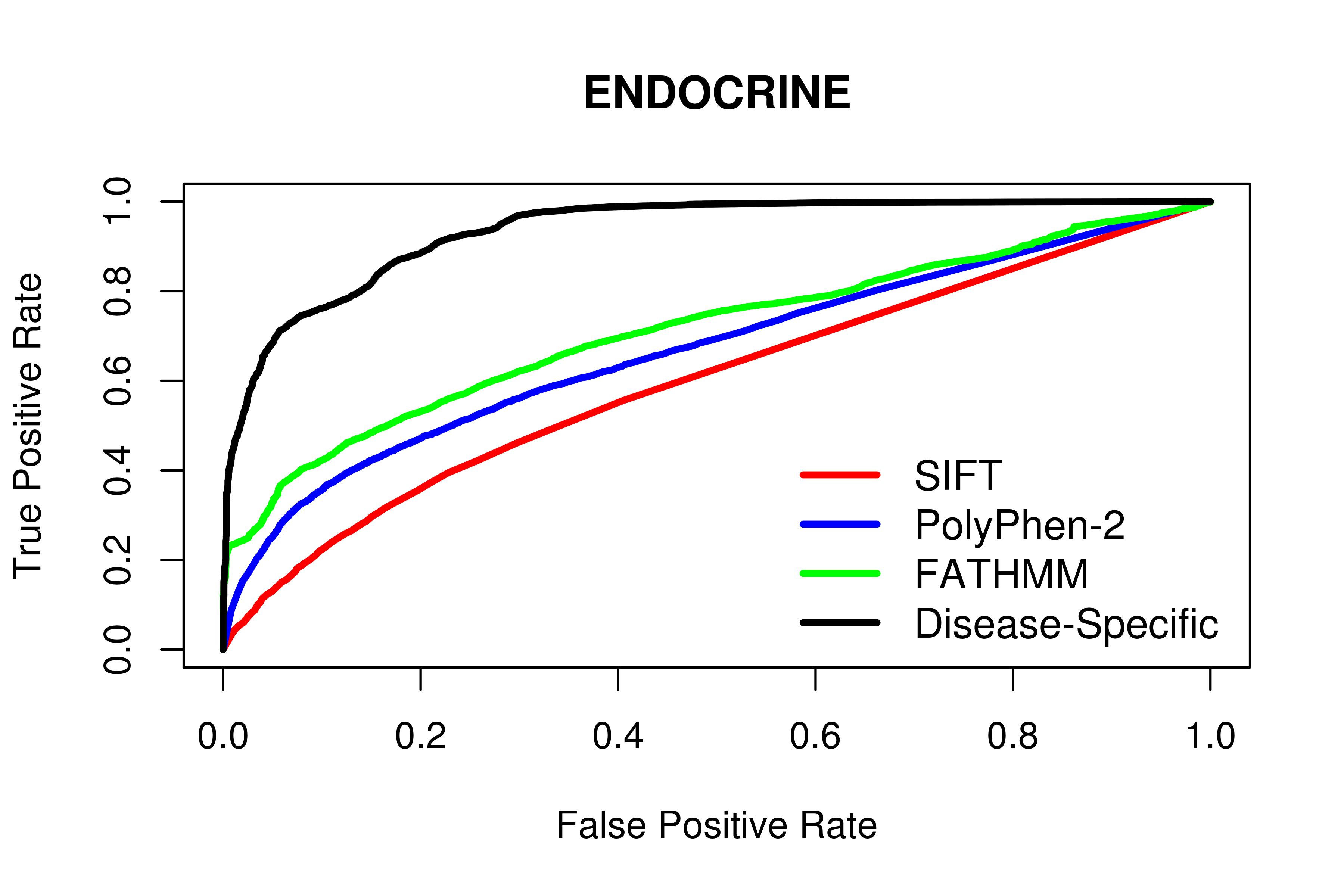


| Algorithm | tp | fp | tn | fn | Accuracy | Precision | Specificity | Sensitivity | NPV | MCC | AUC |
| --- | --- | --- | --- | --- | --- | --- | --- | --- | --- | --- | --- |
| SIFT | 3084 | 39347 | 23443 | 824 | 0.58 | 0.56 | 0.37 | 0.79 | 0.64 | 0.18 | 0.6 |
| PolyPhen-2 | 2890 | 46435 | 35031 | 542 | 0.64 | 0.6 | 0.43 | 0.84 | 0.73 | 0.3 | 0.67 |
| FATHMM | 3597 | 49466 | 33522 | 316 | 0.66 | 0.61 | 0.4 | 0.92 | 0.83 | 0.38 | 0.71 |
| Disease-Specific | 2392 | 1015 | 81973 | 1521 | 0.8 | 0.98 | 0.99 | 0.61 | 0.72 | 0.65 | 0.94 |
| Disease-Specific (20-Fold) | - | - | - | - | 0.79 | 0.97 | 0.98 | 0.6 | 0.71 | 0.63 | - |

In the above, *tp*, *fp*, *tn* and *fn* refer to the number of true positive, false positive, true negative and false negatives observed. *Accuracy*, *Precision*, *Specificity*, *Sensitivity*, Negative Predictive Value (*NPV*) and Matthew’s Correlation Coefficient (*MCC*) were calculated using normalized numbers.

Supp. Info 8. Performance of computational prediction algorithms when tasked with discriminating between “Eye” disease mutations and other disease-causing mutations/putative neutral polymorphisms.


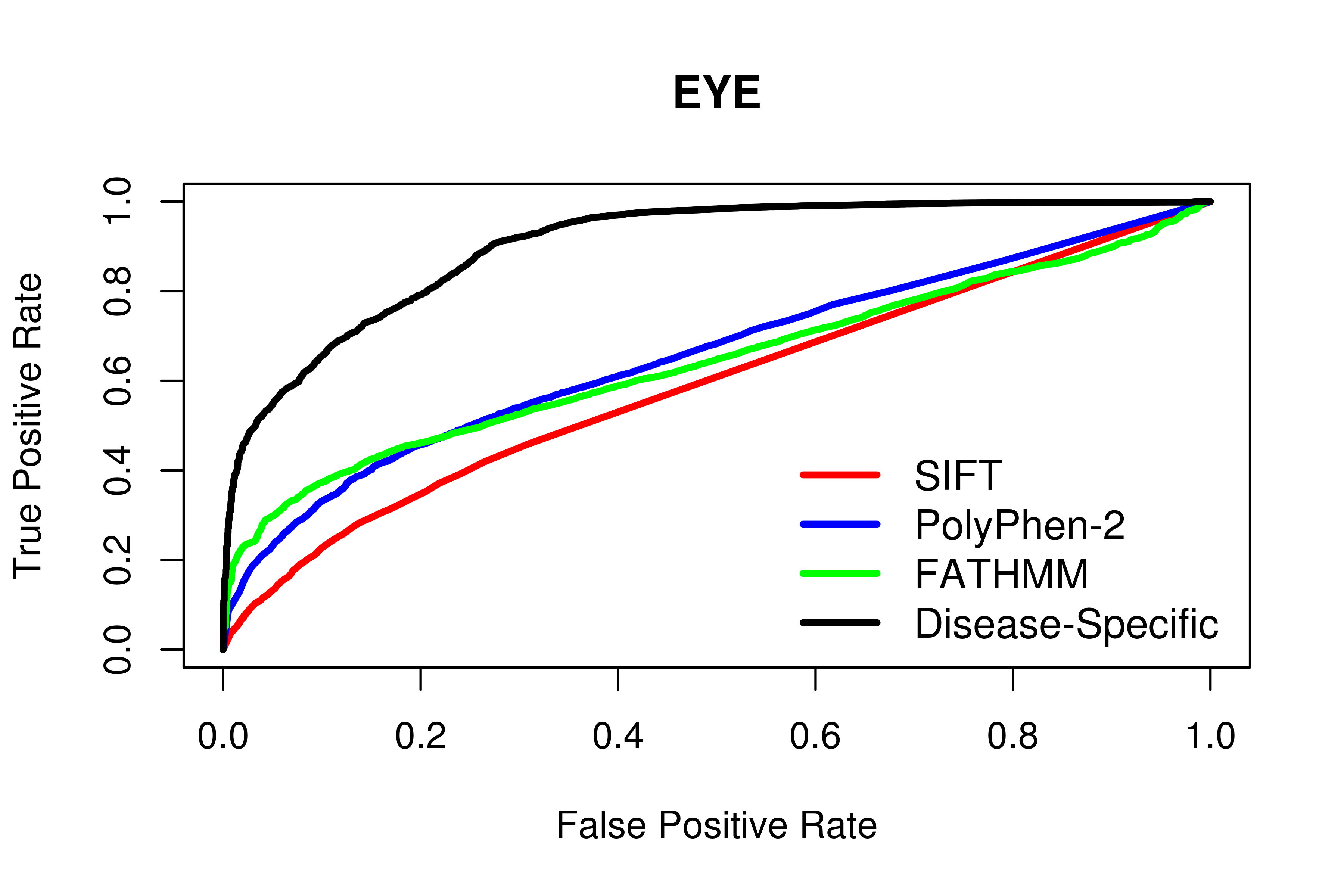


| Algorithm | tp | fp | tn | fn | Accuracy | Precision | Specificity | Sensitivity | NPV | MCC | AUC |
| --- | --- | --- | --- | --- | --- | --- | --- | --- | --- | --- | --- |
| SIFT | 2362 | 40069 | 23607 | 660 | 0.58 | 0.55 | 0.37 | 0.78 | 0.63 | 0.17 | 0.59 |
| PolyPhen-2 | 2472 | 46853 | 35055 | 518 | 0.63 | 0.59 | 0.43 | 0.83 | 0.71 | 0.28 | 0.66 |
| FATHMM | 2701 | 51737 | 32134 | 329 | 0.64 | 0.59 | 0.38 | 0.89 | 0.78 | 0.32 | 0.64 |
| Disease-Specific | 1433 | 1127 | 82744 | 1597 | 0.73 | 0.97 | 0.99 | 0.47 | 0.65 | 0.54 | 0.9 |
| Disease-Specific (20-Fold) | - | - | - | - | 0.72 | 0.96 | 0.98 | 0.45 | 0.64 | 0.51 | - |

In the above, *tp*, *fp*, *tn* and *fn* refer to the number of true positive, false positive, true negative and false negatives observed. *Accuracy*, *Precision*, *Specificity*, *Sensitivity*, Negative Predictive Value (*NPV*) and Matthew’s Correlation Coefficient (*MCC*) were calculated using normalized numbers.

Supp. Info 9. Performance of computational prediction algorithms when tasked with discriminating between “Genitourinary” disease mutations and other disease-causing mutations/putative neutral polymorphisms.


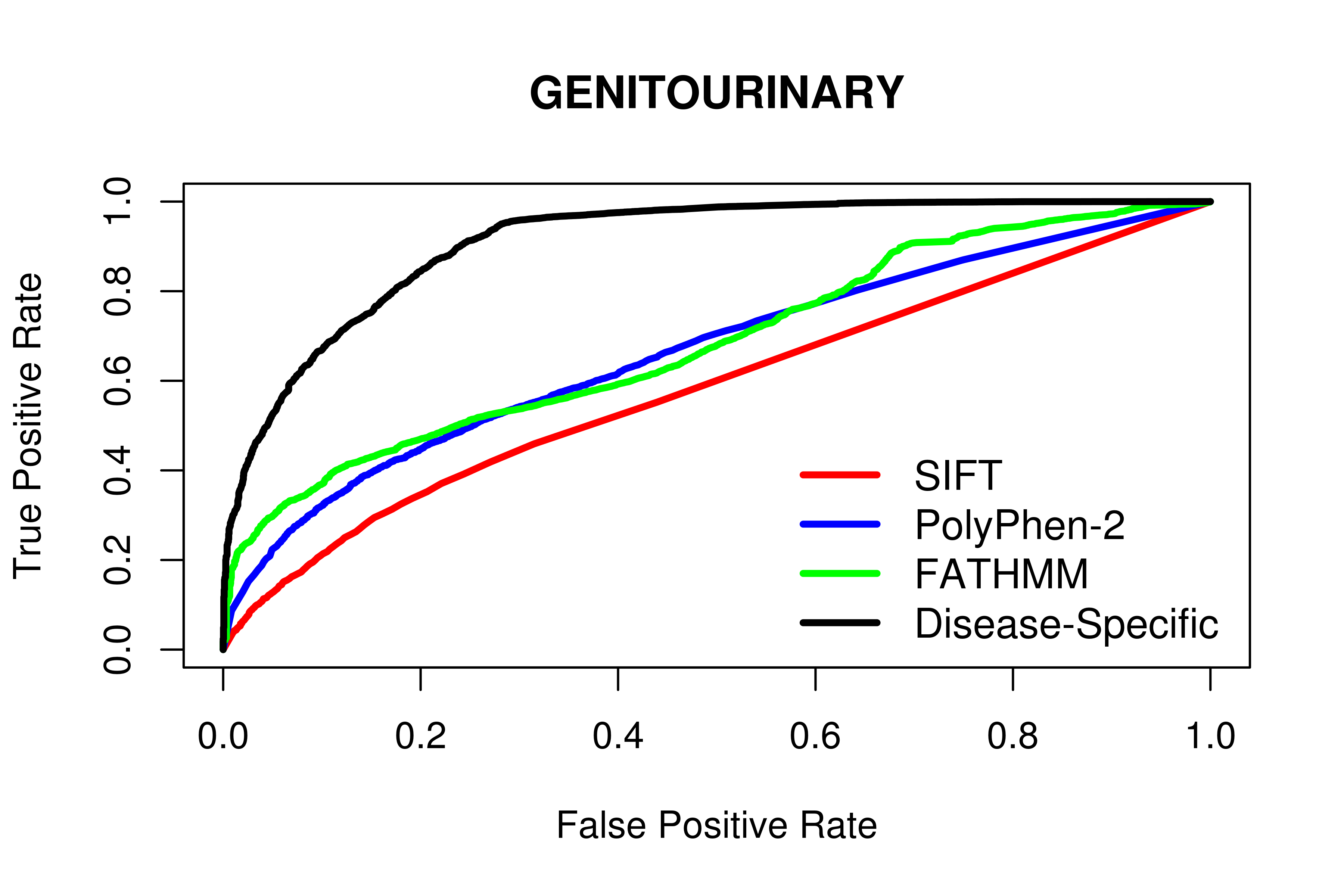


| Algorithm | tp | fp | tn | fn | Accuracy | Precision | Specificity | Sensitivity | NPV | MCC | AUC |
| --- | --- | --- | --- | --- | --- | --- | --- | --- | --- | --- | --- |
| SIFT | 2352 | 40079 | 23600 | 667 | 0.57 | 0.55 | 0.37 | 0.78 | 0.63 | 0.16 | 0.59 |
| PolyPhen-2 | 2214 | 47111 | 35083 | 490 | 0.62 | 0.59 | 0.43 | 0.82 | 0.7 | 0.27 | 0.66 |
| FATHMM | 2661 | 49707 | 34164 | 369 | 0.64 | 0.6 | 0.41 | 0.88 | 0.77 | 0.32 | 0.68 |
| Disease-Specific | 1601 | 1327 | 82544 | 1429 | 0.76 | 0.97 | 0.98 | 0.53 | 0.68 | 0.58 | 0.91 |
| Disease-Specific (20-Fold) | - | - | - | - | 0.74 | 0.96 | 0.98 | 0.5 | 0.66 | 0.55 | - |

In the above, *tp*, *fp*, *tn* and *fn* refer to the number of true positive, false positive, true negative and false negatives observed. *Accuracy*, *Precision*, *Specificity*, *Sensitivity*, Negative Predictive Value (*NPV*) and Matthew’s Correlation Coefficient (*MCC*) were calculated using normalized numbers.

Supp. Info 10. Performance of computational prediction algorithms when tasked with discriminating between “Heart” disease mutations and other disease-causing mutations/putative neutral polymorphisms.


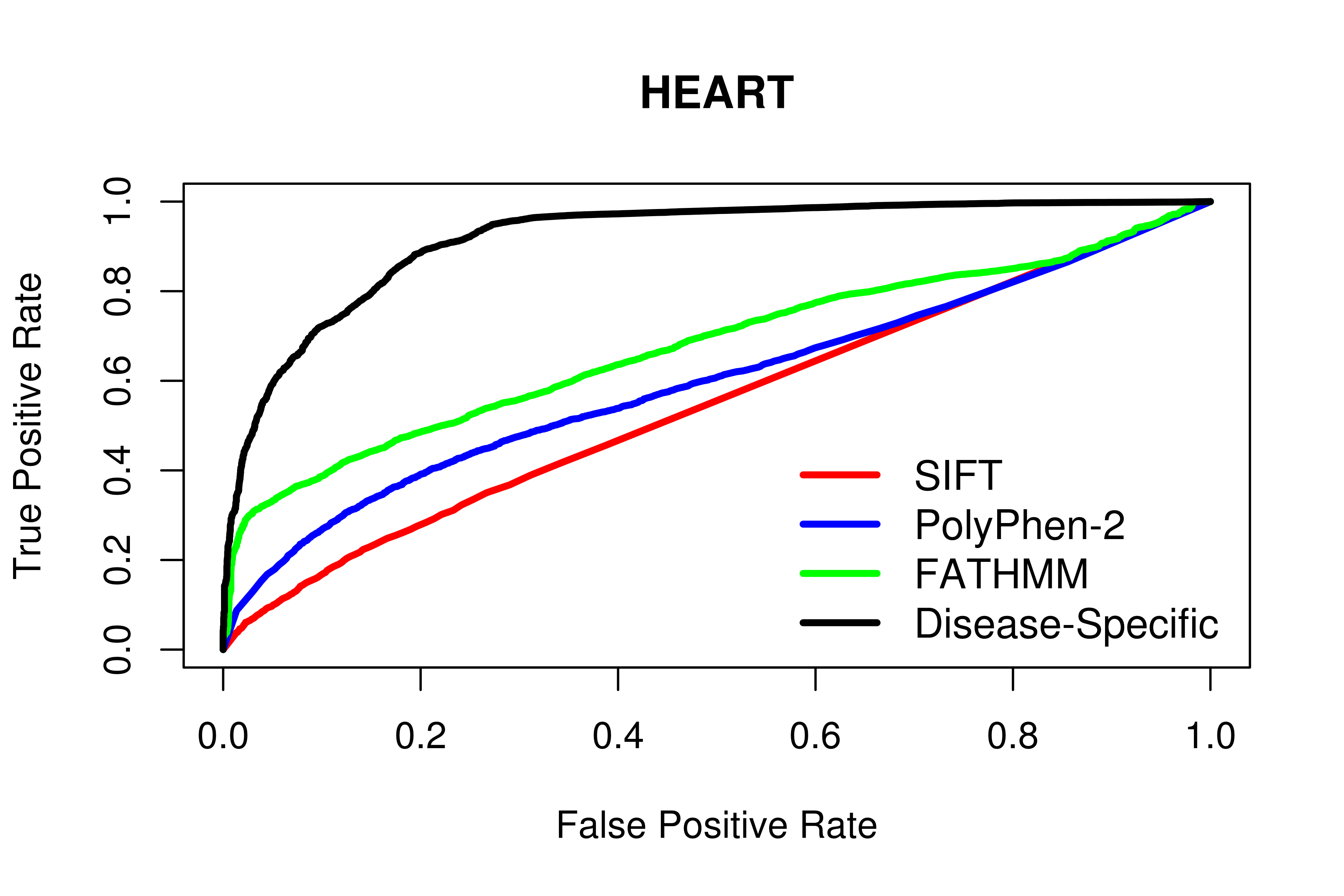


| Algorithm | tp | fp | tn | fn | Accuracy | Precision | Specificity | Sensitivity | NPV | MCC | AUC |
| --- | --- | --- | --- | --- | --- | --- | --- | --- | --- | --- | --- |
| SIFT | 2422 | 40009 | 23276 | 991 | 0.54 | 0.53 | 0.37 | 0.71 | 0.56 | 0.08 | 0.55 |
| PolyPhen-2 | 2683 | 46642 | 34736 | 837 | 0.59 | 0.57 | 0.43 | 0.76 | 0.64 | 0.2 | 0.6 |
| FATHMM | 3588 | 56453 | 26706 | 154 | 0.64 | 0.59 | 0.32 | 0.96 | 0.89 | 0.36 | 0.68 |
| Disease-Specific | 2536 | 2885 | 80274 | 1206 | 0.82 | 0.95 | 0.97 | 0.68 | 0.75 | 0.67 | 0.92 |
| Disease-Specific (20-Fold) | - | - | - | - | 0.8 | 0.91 | 0.93 | 0.66 | 0.73 | 0.62 | - |

In the above, *tp*, *fp*, *tn* and *fn* refer to the number of true positive, false positive, true negative and false negatives observed. *Accuracy*, *Precision*, *Specificity*, *Sensitivity*, Negative Predictive Value (*NPV*) and Matthew’s Correlation Coefficient (*MCC*) were calculated using normalized numbers.

Supp. Info 11. Performance of computational prediction algorithms when tasked with discriminating between “Immune” disease mutations and other disease-causing mutations/putative neutral polymorphisms.


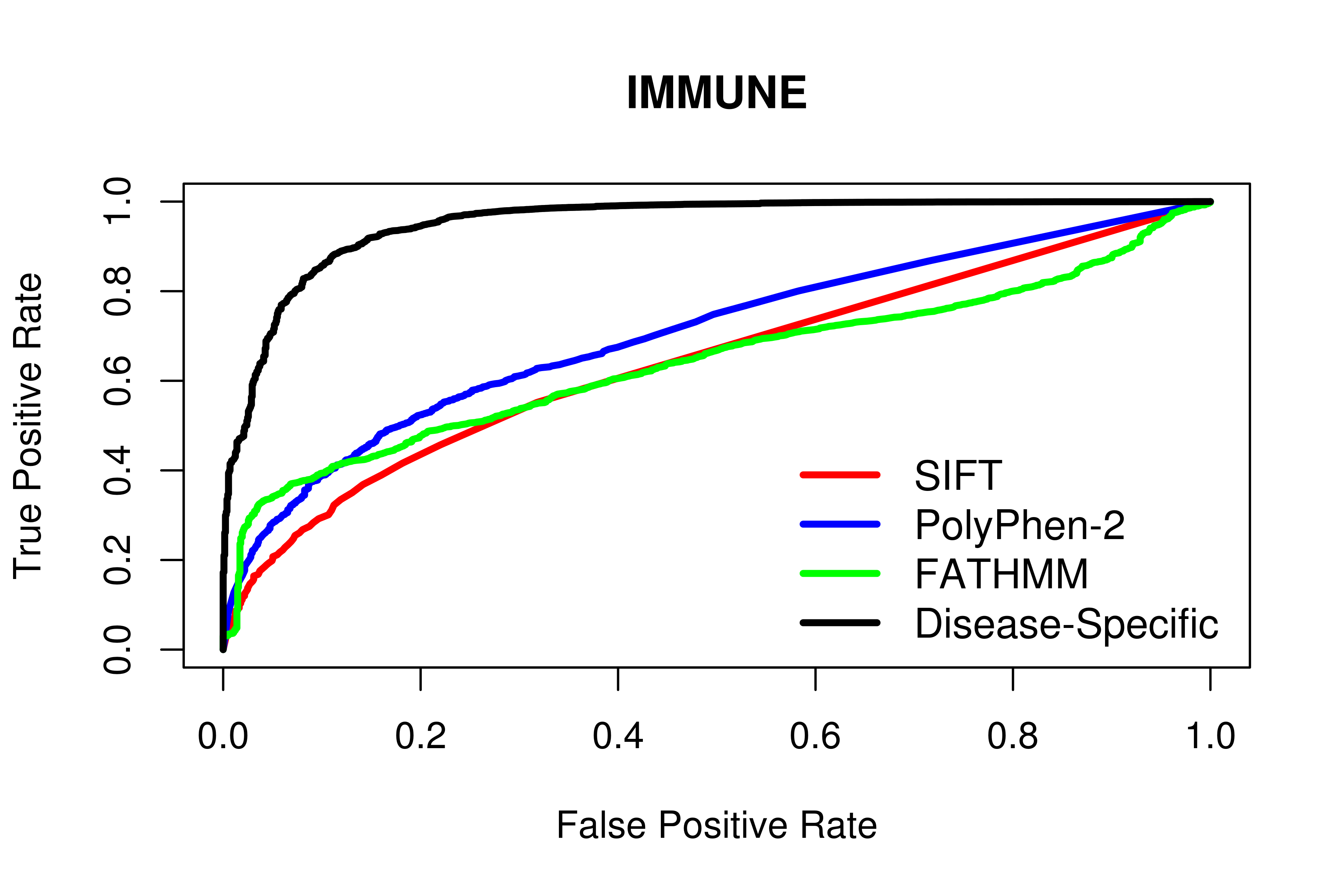


| Algorithm | tp | fp | tn | fn | Accuracy | Precision | Specificity | Sensitivity | NPV | MCC | AUC |
| --- | --- | --- | --- | --- | --- | --- | --- | --- | --- | --- | --- |
| SIFT | 1110 | 41321 | 24084 | 183 | 0.61 | 0.58 | 0.37 | 0.86 | 0.72 | 0.26 | 0.64 |
| PolyPhen-2 | 1125 | 48200 | 35413 | 160 | 0.65 | 0.6 | 0.42 | 0.88 | 0.77 | 0.34 | 0.7 |
| FATHMM | 1046 | 45596 | 40012 | 247 | 0.64 | 0.6 | 0.47 | 0.81 | 0.71 | 0.29 | 0.64 |
| Disease-Specific | 590 | 385 | 85223 | 703 | 0.73 | 0.99 | 1 | 0.46 | 0.65 | 0.54 | 0.95 |
| Disease-Specific (20-Fold) | - | - | - | - | 0.72 | 0.99 | 0.99 | 0.44 | 0.64 | 0.52 | - |

In the above, *tp*, *fp*, *tn* and *fn* refer to the number of true positive, false positive, true negative and false negatives observed. *Accuracy*, *Precision*, *Specificity*, *Sensitivity*, Negative Predictive Value (*NPV*) and Matthew’s Correlation Coefficient (*MCC*) were calculated using normalized numbers.

Supp. Info 12. Performance of computational prediction algorithms when tasked with discriminating between “Metabolic” disease mutations and other disease-causing mutations/putative neutral polymorphisms.


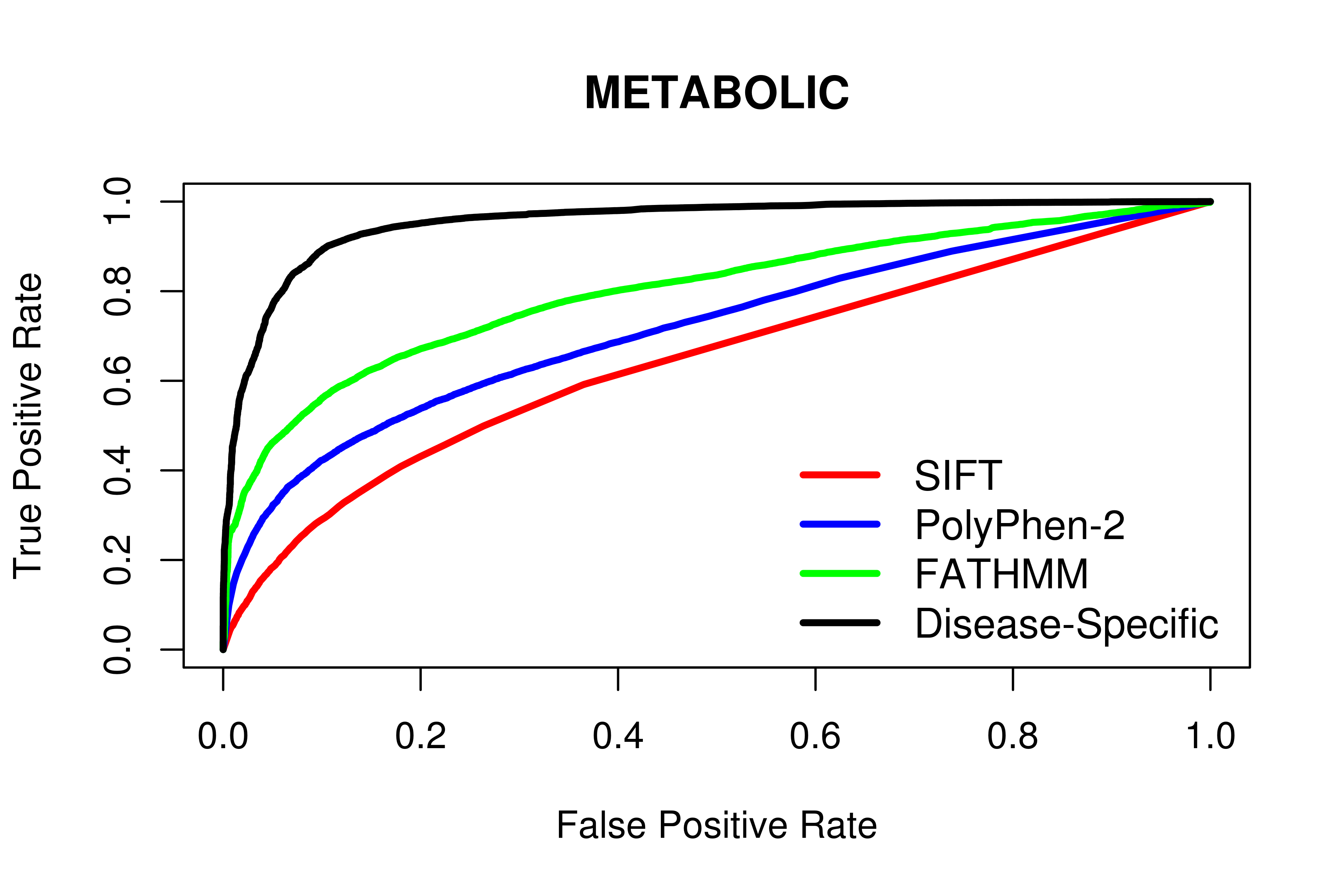


| Algorithm | tp | fp | tn | fn | Accuracy | Precision | Specificity | Sensitivity | NPV | MCC | AUC |
| --- | --- | --- | --- | --- | --- | --- | --- | --- | --- | --- | --- |
| SIFT | 10731 | 31700 | 21913 | 2354 | 0.61 | 0.58 | 0.41 | 0.82 | 0.69 | 0.25 | 0.64 |
| PolyPhen-2 | 11337 | 37988 | 33788 | 1785 | 0.67 | 0.62 | 0.47 | 0.86 | 0.78 | 0.36 | 0.72 |
| FATHMM | 13068 | 39914 | 33271 | 648 | 0.7 | 0.64 | 0.45 | 0.95 | 0.91 | 0.47 | 0.8 |
| Disease-Specific | 10767 | 3209 | 69976 | 2949 | 0.87 | 0.95 | 0.96 | 0.78 | 0.82 | 0.75 | 0.95 |
| Disease-Specific (20-Fold) | - | - | - | - | 0.86 | 0.94 | 0.95 | 0.77 | 0.81 | 0.74 | - |

In the above, *tp*, *fp*, *tn* and *fn* refer to the number of true positive, false positive, true negative and false negatives observed. *Accuracy*, *Precision*, *Specificity*, *Sensitivity*, Negative Predictive Value (*NPV*) and Matthew’s Correlation Coefficient (*MCC*) were calculated using normalized numbers.

Supp. Info 13. Performance of computational prediction algorithms when tasked with discriminating between “Musculoskeletal” disease mutations and other disease-causing mutations/putative neutral polymorphisms.


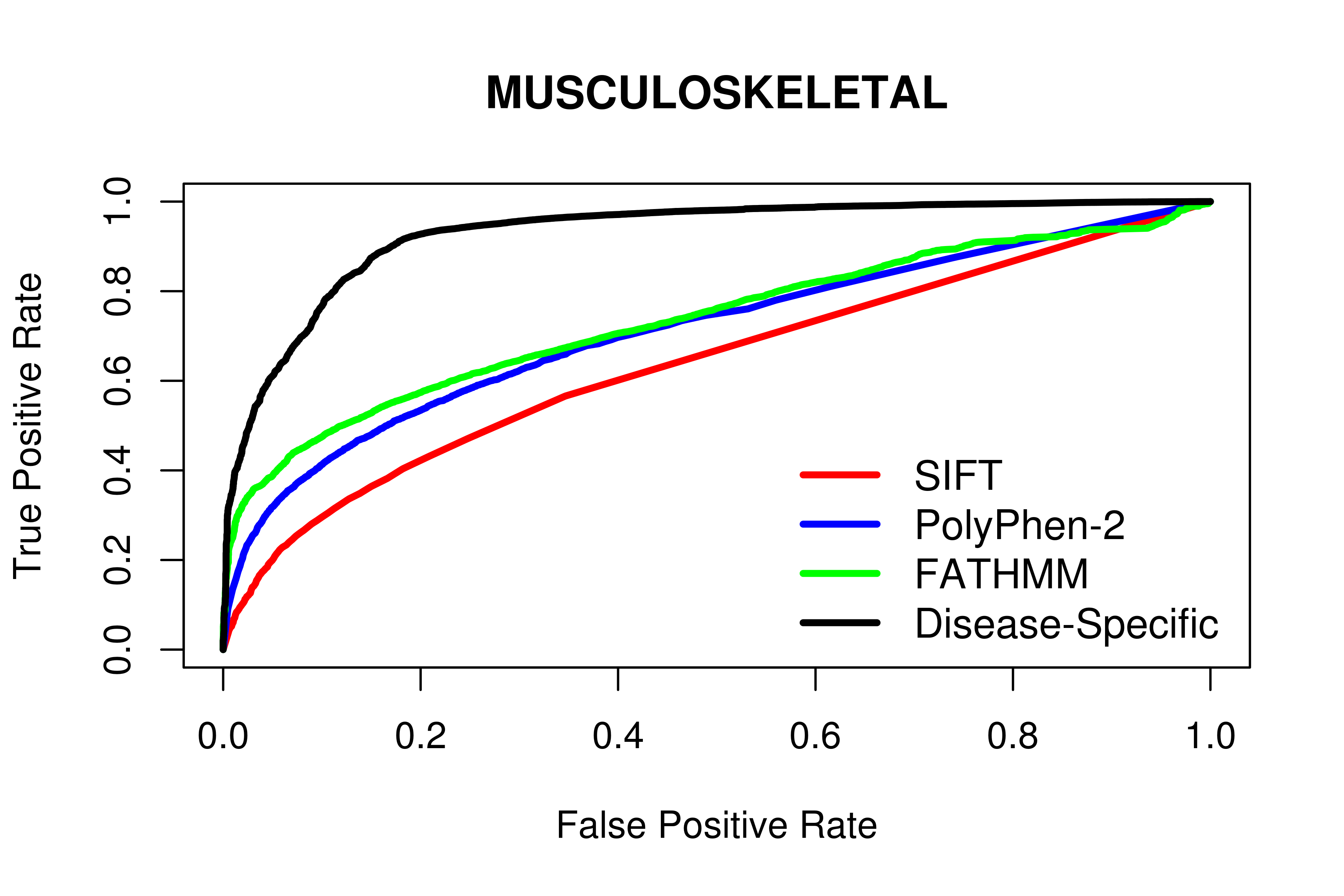


| Algorithm | tp | fp | tn | fn | Accuracy | Precision | Specificity | Sensitivity | NPV | MCC | AUC |
| --- | --- | --- | --- | --- | --- | --- | --- | --- | --- | --- | --- |
| SIFT | 4730 | 37701 | 23323 | 944 | 0.61 | 0.57 | 0.38 | 0.83 | 0.7 | 0.24 | 0.64 |
| PolyPhen-2 | 5278 | 44047 | 34859 | 714 | 0.66 | 0.61 | 0.44 | 0.88 | 0.79 | 0.36 | 0.71 |
| FATHMM | 5902 | 51596 | 29202 | 201 | 0.66 | 0.6 | 0.36 | 0.97 | 0.92 | 0.41 | 0.73 |
| Disease-Specific | 4120 | 3123 | 77675 | 1983 | 0.82 | 0.95 | 0.96 | 0.68 | 0.75 | 0.66 | 0.93 |
| Disease-Specific (20-Fold) | - | - | - | - | 0.8 | 0.92 | 0.94 | 0.66 | 0.74 | 0.63 | - |

In the above, *tp*, *fp*, *tn* and *fn* refer to the number of true positive, false positive, true negative and false negatives observed. *Accuracy*, *Precision*, *Specificity*, *Sensitivity*, Negative Predictive Value (*NPV*) and Matthew’s Correlation Coefficient (*MCC*) were calculated using normalized numbers.

Supp. Info 14. Performance of computational prediction algorithms when tasked with discriminating between “Nervous System” disease mutations and other disease-causing mutations/putative neutral polymorphisms.


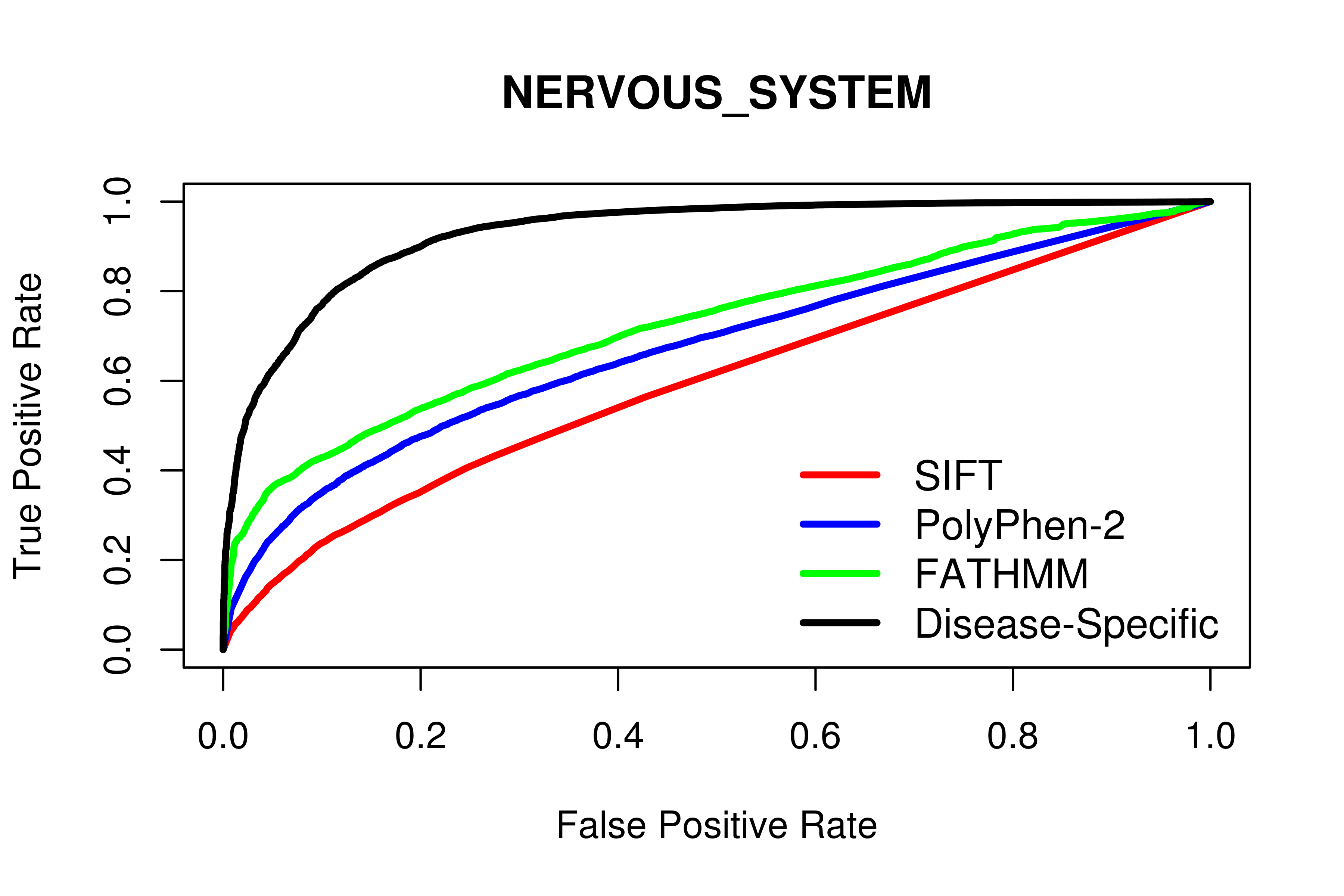


| Algorithm | tp | fp | tn | fn | Accuracy | Precision | Specificity | Sensitivity | NPV | MCC | AUC |
| --- | --- | --- | --- | --- | --- | --- | --- | --- | --- | --- | --- |
| SIFT | 6550 | 35881 | 22344 | 1923 | 0.58 | 0.56 | 0.38 | 0.77 | 0.63 | 0.17 | 0.6 |
| PolyPhen-2 | 6672 | 42653 | 34182 | 1391 | 0.64 | 0.6 | 0.44 | 0.83 | 0.72 | 0.29 | 0.67 |
| FATHMM | 7939 | 47844 | 30506 | 612 | 0.66 | 0.6 | 0.39 | 0.93 | 0.84 | 0.38 | 0.72 |
| Disease-Specific | 5560 | 2419 | 75931 | 2991 | 0.81 | 0.95 | 0.97 | 0.65 | 0.73 | 0.65 | 0.93 |
| Disease-Specific (20-Fold) | - | - | - | - | 0.8 | 0.94 | 0.96 | 0.63 | 0.72 | 0.63 | - |

In the above, *tp*, *fp*, *tn* and *fn* refer to the number of true positive, false positive, true negative and false negatives observed. *Accuracy*, *Precision*, *Specificity*, *Sensitivity*, Negative Predictive Value (*NPV*) and Matthew’s Correlation Coefficient (*MCC*) were calculated using normalized numbers.

Supp. Info 15. Performance of computational prediction algorithms when tasked with discriminating between “Psychiatric” disease mutations and other disease-causing mutations/putative neutral polymorphisms.


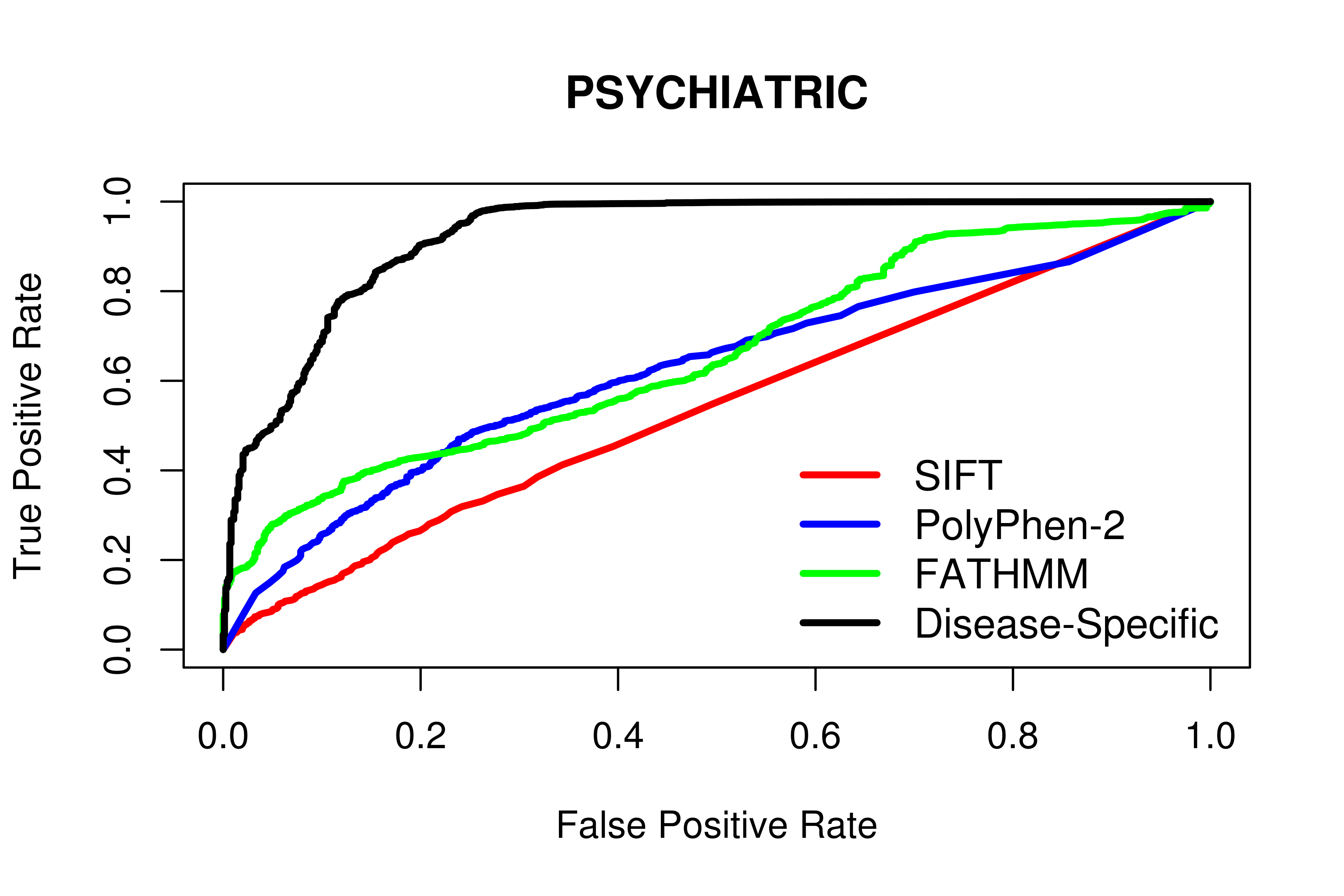


| Algorithm | tp | fp | tn | fn | Accuracy | Precision | Specificity | Sensitivity | NPV | MCC | AUC |
| --- | --- | --- | --- | --- | --- | --- | --- | --- | --- | --- | --- |
| SIFT | 500 | 41931 | 24048 | 219 | 0.53 | 0.52 | 0.36 | 0.7 | 0.54 | 0.06 | 0.54 |
| PolyPhen-2 | 571 | 48754 | 35418 | 155 | 0.6 | 0.58 | 0.42 | 0.79 | 0.66 | 0.22 | 0.62 |
| FATHMM | 504 | 42672 | 43483 | 242 | 0.59 | 0.58 | 0.5 | 0.68 | 0.61 | 0.18 | 0.66 |
| Disease-Specific | 379 | 165 | 85990 | 367 | 0.75 | 1 | 1 | 0.51 | 0.67 | 0.58 | 0.92 |
| Disease-Specific (20-Fold) | - | - | - | - | 0.74 | 0.99 | 1 | 0.48 | 0.66 | 0.56 | - |

In the above, *tp*, *fp*, *tn* and *fn* refer to the number of true positive, false positive, true negative and false negatives observed. *Accuracy*, *Precision*, *Specificity*, *Sensitivity*, Negative Predictive Value (*NPV*) and Matthew’s Correlation Coefficient (*MCC*) were calculated using normalized numbers.

Supp. Info 16. Performance of computational prediction algorithms when tasked with discriminating between “Reproductive” disease mutations and other disease-causing mutations/putative neutral polymorphisms.


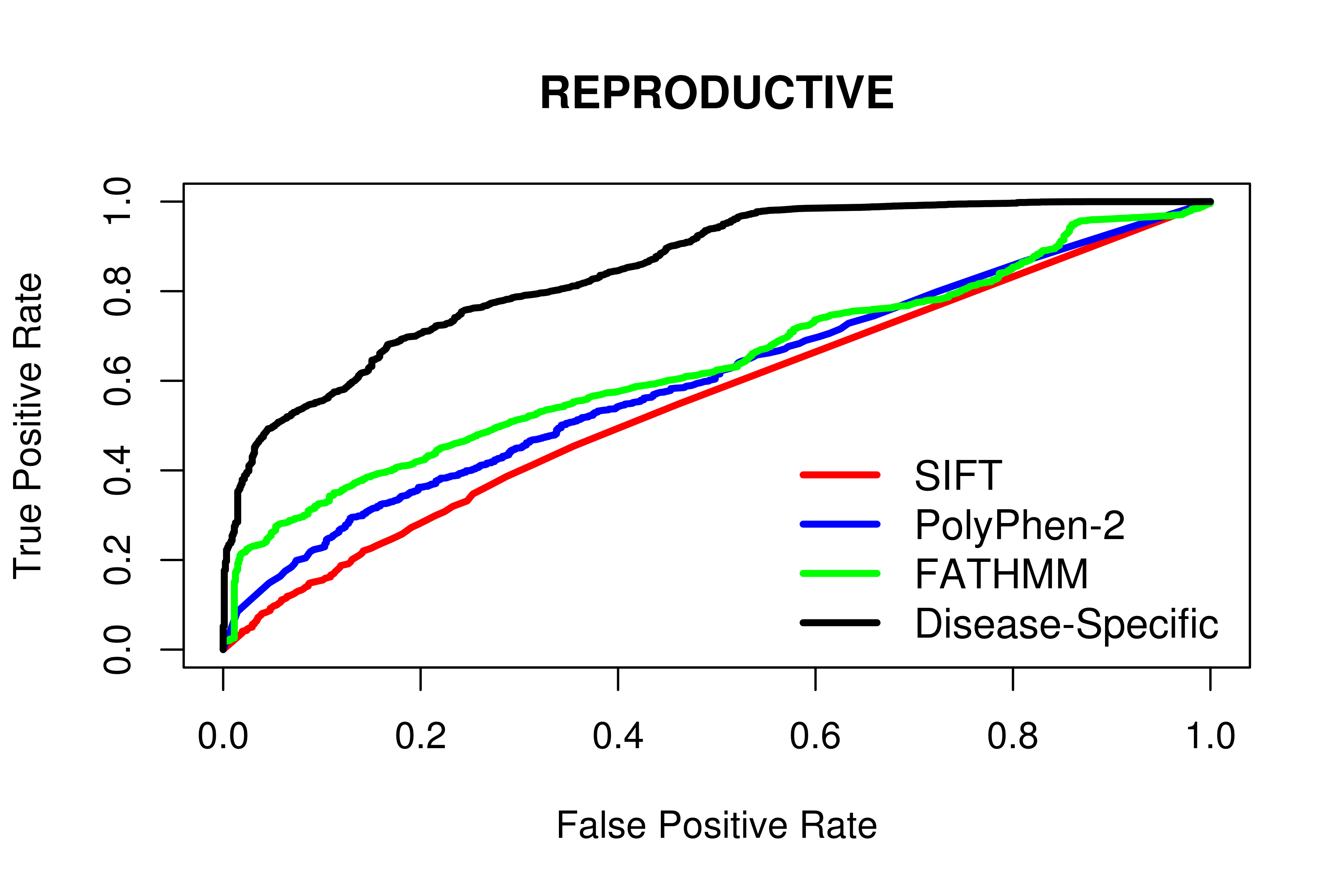


| Algorithm | tp | fp | tn | fn | Accuracy | Precision | Specificity | Sensitivity | NPV | MCC | AUC |
| --- | --- | --- | --- | --- | --- | --- | --- | --- | --- | --- | --- |
| SIFT | 645 | 41786 | 24030 | 237 | 0.55 | 0.54 | 0.37 | 0.73 | 0.58 | 0.1 | 0.56 |
| PolyPhen-2 | 451 | 48874 | 35402 | 171 | 0.57 | 0.56 | 0.42 | 0.73 | 0.6 | 0.15 | 0.6 |
| FATHMM | 370 | 24336 | 61682 | 513 | 0.57 | 0.6 | 0.72 | 0.42 | 0.55 | 0.14 | 0.64 |
| Disease-Specific | 131 | 36 | 85982 | 752 | 0.57 | 1 | 1 | 0.15 | 0.54 | 0.28 | 0.85 |
| Disease-Specific (20-Fold) | - | - | - | - | 0.57 | 0.99 | 1 | 0.14 | 0.54 | 0.26 | - |

In the above, *tp*, *fp*, *tn* and *fn* refer to the number of true positive, false positive, true negative and false negatives observed. *Accuracy*, *Precision*, *Specificity*, *Sensitivity*, Negative Predictive Value (*NPV*) and Matthew’s Correlation Coefficient (*MCC*) were calculated using normalized numbers.

Supp. Info 17. Performance of computational prediction algorithms when tasked with discriminating between “Respiratory” disease mutations and other disease-causing mutations/putative neutral polymorphisms.


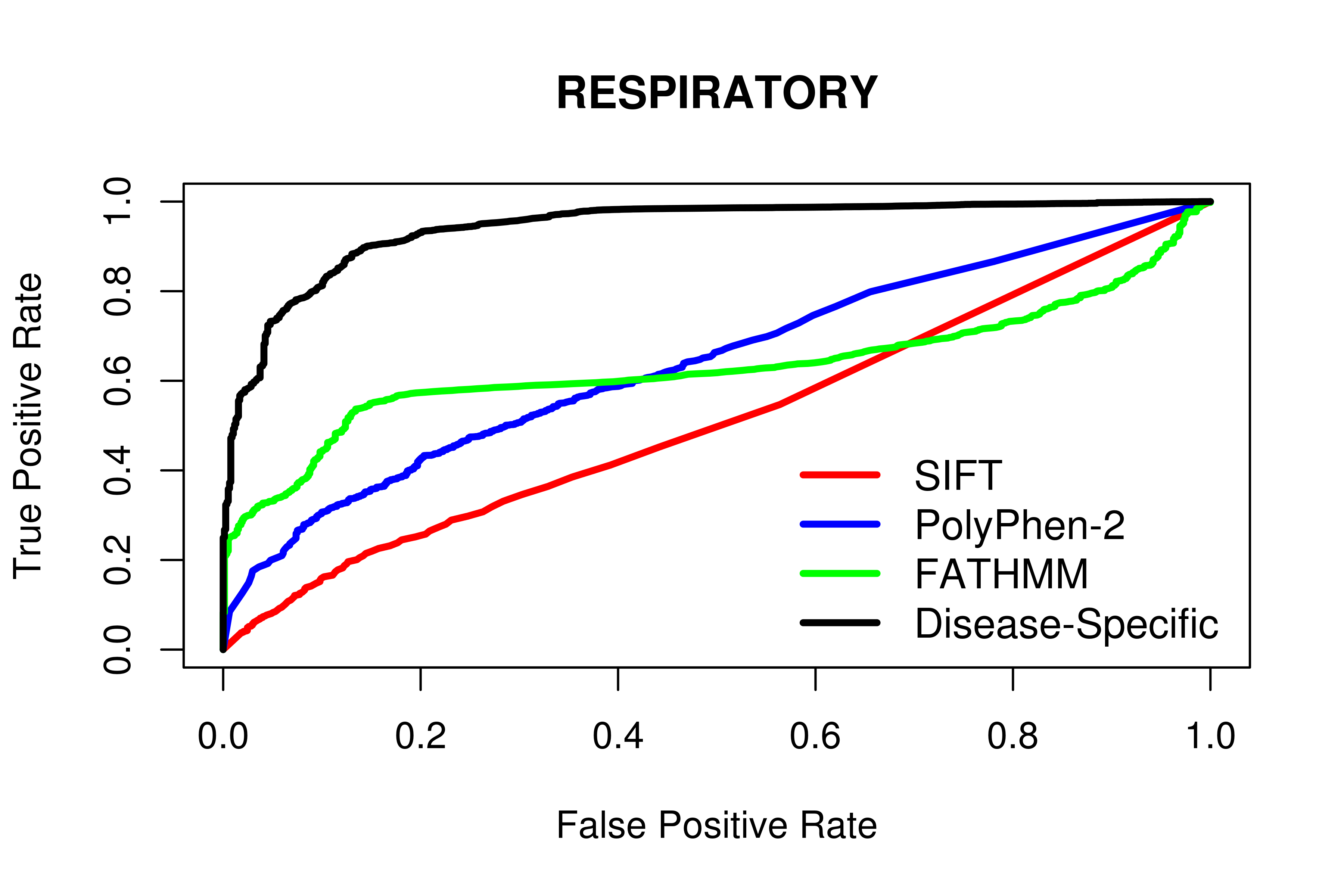


| Algorithm | tp | fp | tn | fn | Accuracy | Precision | Specificity | Sensitivity | NPV | MCC | AUC |
| --- | --- | --- | --- | --- | --- | --- | --- | --- | --- | --- | --- |
| SIFT | 520 | 41911 | 24012 | 255 | 0.52 | 0.51 | 0.36 | 0.67 | 0.53 | 0.04 | 0.51 |
| PolyPhen-2 | 619 | 48706 | 35420 | 153 | 0.61 | 0.58 | 0.42 | 0.8 | 0.68 | 0.24 | 0.64 |
| FATHMM | 758 | 60581 | 25545 | 17 | 0.64 | 0.58 | 0.3 | 0.98 | 0.93 | 0.38 | 0.63 |
| Disease-Specific | 478 | 1604 | 84522 | 297 | 0.8 | 0.97 | 0.98 | 0.62 | 0.72 | 0.64 | 0.94 |
| Disease-Specific (20-Fold) | - | - | - | - | 0.78 | 0.92 | 0.95 | 0.6 | 0.71 | 0.59 | - |

In the above, *tp*, *fp*, *tn* and *fn* refer to the number of true positive, false positive, true negative and false negatives observed. *Accuracy*, *Precision*, *Specificity*, *Sensitivity*, Negative Predictive Value (*NPV*) and Matthew’s Correlation Coefficient (*MCC*) were calculated using normalized numbers.

Supp. Info 18. Performance of computational prediction algorithms when tasked with discriminating between “Skin” disease mutations and other disease-causing mutations/putative neutral polymorphisms.


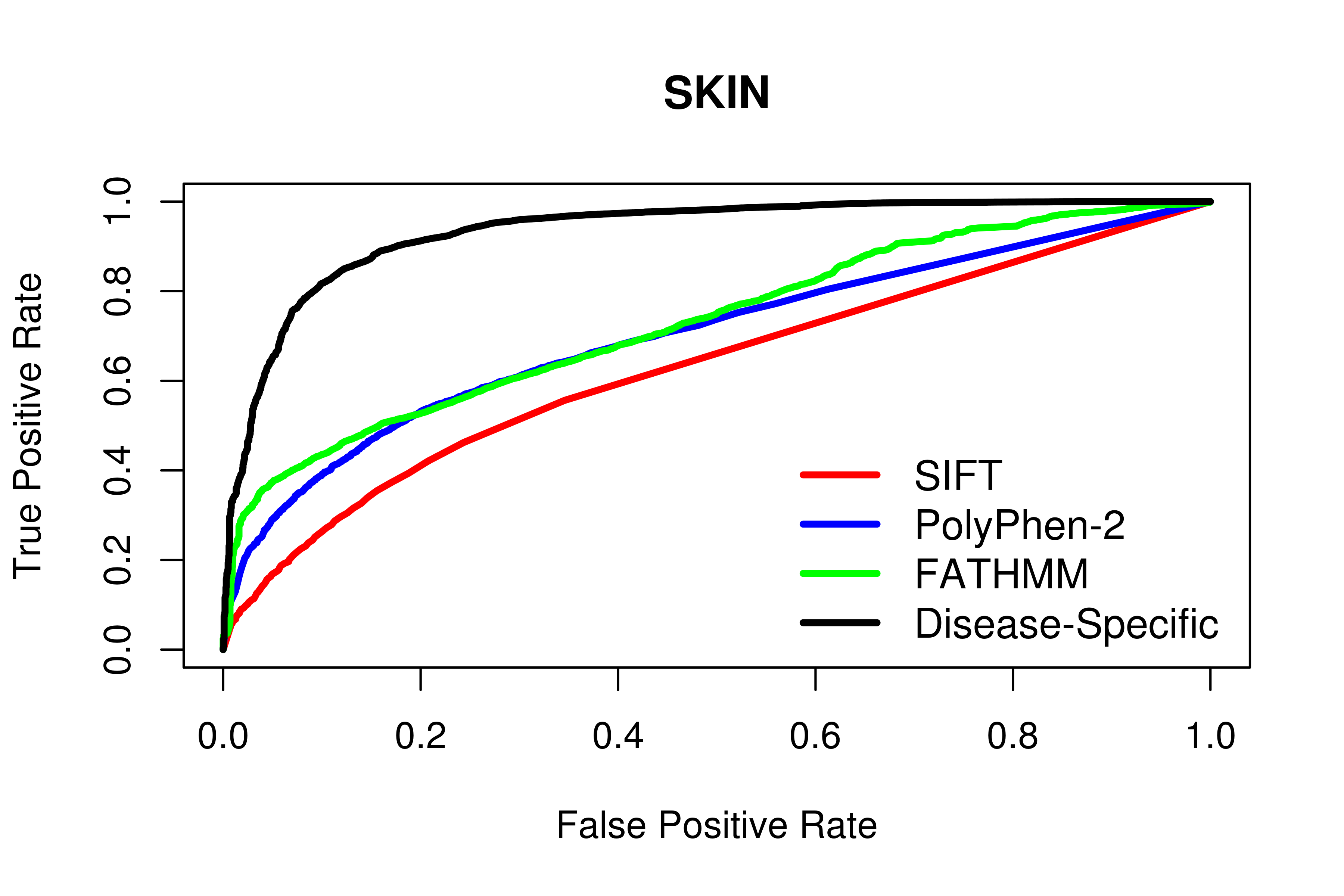


| Algorithm | tp | fp | tn | fn | Accuracy | Precision | Specificity | Sensitivity | NPV | MCC | AUC |
| --- | --- | --- | --- | --- | --- | --- | --- | --- | --- | --- | --- |
| SIFT | 2425 | 40006 | 23770 | 497 | 0.6 | 0.57 | 0.37 | 0.83 | 0.69 | 0.23 | 0.63 |
| PolyPhen-2 | 2675 | 46650 | 35188 | 385 | 0.65 | 0.61 | 0.43 | 0.87 | 0.77 | 0.34 | 0.7 |
| FATHMM | 2727 | 43148 | 40570 | 456 | 0.67 | 0.62 | 0.48 | 0.86 | 0.77 | 0.37 | 0.73 |
| Disease-Specific | 1230 | 537 | 83181 | 1953 | 0.69 | 0.98 | 0.99 | 0.39 | 0.62 | 0.48 | 0.93 |
| Disease-Specific (20-Fold) | - | - | - | - | 0.68 | 0.98 | 0.99 | 0.37 | 0.61 | 0.46 | - |

In the above, *tp*, *fp*, *tn* and *fn* refer to the number of true positive, false positive, true negative and false negatives observed. *Accuracy*, *Precision*, *Specificity*, *Sensitivity*, Negative Predictive Value (*NPV*) and Matthew’s Correlation Coefficient (*MCC*) were calculated using normalized numbers.

**Supp. Info 19.** Performance of our Disease-Specific Approach and Naive Weighting Scheme when using Balanced and Imbalanced Weights (Inherited Disease Mutations).

| Algorithm | tp | fp | tn | fn | Accuracy | Precision | Specificity | Sensitivity | NPV | MCC |
| --- | --- | --- | --- | --- | --- | --- | --- | --- | --- | --- |
| Disease-Specific (All) | 44330 | 7007 | 29931 | 5633 | 0.85 | 0.82 | 0.81 | 0.89 | 0.88 | 0.7 |
| Naive (All) | 46103 | 10286 | 26652 | 3860 | 0.82 | 0.77 | 0.72 | 0.92 | 0.9 | 0.66 |
| Disease-Specific (5) | 3962 | 2271 | 10748 | 669 | 0.84 | 0.83 | 0.83 | 0.86 | 0.85 | 0.68 |
| Naive (5) | 3176 | 1550 | 11469 | 1455 | 0.78 | 0.85 | 0.88 | 0.69 | 0.74 | 0.58 |
| Disease-Specific (10) | 6565 | 3235 | 15058 | 1008 | 0.85 | 0.83 | 0.82 | 0.87 | 0.86 | 0.69 |
| Naive (10) | 5678 | 2464 | 15829 | 1895 | 0.81 | 0.85 | 0.87 | 0.75 | 0.78 | 0.62 |
| Disease-Specific (25) | 12432 | 4923 | 19122 | 1450 | 0.85 | 0.81 | 0.8 | 0.9 | 0.88 | 0.69 |
| Naive (52) | 11405 | 4096 | 19949 | 2477 | 0.83 | 0.83 | 0.83 | 0.82 | 0.82 | 0.65 |
| Disease-Specific (100) | 27481 | 8148 | 22144 | 2159 | 0.83 | 0.78 | 0.73 | 0.93 | 0.91 | 0.67 |
| Naive (100) | 26324 | 7292 | 23000 | 3316 | 0.82 | 0.79 | 0.76 | 0.89 | 0.87 | 0.65 |
| Disease-Specific (250) | 36302 | 9492 | 23019 | 2231 | 0.83 | 0.76 | 0.71 | 0.94 | 0.92 | 0.67 |
| Naive (250) | 35121 | 8630 | 23881 | 3412 | 0.82 | 0.77 | 0.73 | 0.91 | 0.89 | 0.66 |

In the above, *tp*, *fp*, *tn* and *fn* refer to the number of true positive, false positive, true negative and false negatives observed. *Accuracy*, *Precision*, *Specificity*, *Sensitivity*, Negative Predictive Value (*NPV*) and Matthew’s Correlation Coefficient (*MCC*) were calculated using normalized numbers. The number shown in brackets refer to the maximum difference between disease-associated mutations and other germline polymorphisms allowed in the weighting scheme; the higher this number is, the more imbalanced the weighting scheme is.

**Supp. Info 20.** Performance of our Disease-Specific Approach and Naive Weighting Scheme when using Balanced and Imbalanced Weights (Blood Disease Mutations).

| Algorithm | tp | fp | tn | fn | Accuracy | Precision | Specificity | Sensitivity | NPV | MCC |
| --- | --- | --- | --- | --- | --- | --- | --- | --- | --- | --- |
| Disease-Specific (All) | 860 | 487 | 84939 | 615 | 0.79 | 0.99 | 0.99 | 0.58 | 0.7 | 0.63 |
| Naive (All) | 886 | 202 | 85224 | 589 | 0.8 | 1 | 1 | 0.6 | 0.71 | 0.65 |
| Disease-Specific (5) | 111 | 373 | 11348 | 40 | 0.85 | 0.96 | 0.97 | 0.74 | 0.79 | 0.72 |
| Naive (5) | 79 | 44 | 11677 | 72 | 0.76 | 0.99 | 1 | 0.52 | 0.68 | 0.59 |
| Disease-Specific (10) | 266 | 498 | 17891 | 83 | 0.87 | 0.97 | 0.97 | 0.76 | 0.8 | 0.75 |
| Naive (10) | 220 | 89 | 18300 | 129 | 0.81 | 0.99 | 1 | 0.63 | 0.73 | 0.67 |
| Disease-Specific (25) | 376 | 622 | 28259 | 179 | 0.83 | 0.97 | 0.98 | 0.68 | 0.75 | 0.69 |
| Naive (52) | 321 | 119 | 28762 | 234 | 0.79 | 0.99 | 1 | 0.58 | 0.7 | 0.63 |
| Disease-Specific (100) | 828 | 797 | 50682 | 384 | 0.83 | 0.98 | 0.98 | 0.68 | 0.76 | 0.7 |
| Naive (100) | 745 | 195 | 51284 | 467 | 0.81 | 0.99 | 1 | 0.61 | 0.72 | 0.66 |
| Disease-Specific (250) | 969 | 905 | 64188 | 430 | 0.84 | 0.98 | 0.99 | 0.69 | 0.76 | 0.71 |
| Naive (250) | 886 | 202 | 64891 | 513 | 0.82 | 1 | 1 | 0.63 | 0.73 | 0.68 |

In the above, *tp*, *fp*, *tn* and *fn* refer to the number of true positive, false positive, true negative and false negatives observed. *Accuracy*, *Precision*, *Specificity*, *Sensitivity*, Negative Predictive Value (*NPV*) and Matthew’s Correlation Coefficient (*MCC*) were calculated using normalized numbers. The number shown in brackets refer to the maximum difference between disease-associated mutations and other germline polymorphisms allowed in the weighting scheme; the higher this number is, the more imbalanced the weighting scheme is.

**Supp. Info 21.** Performance of our Disease-Specific Approach and Naive Weighting Scheme when using Balanced and Imbalanced Weights (Blood Coagulation Disease Mutations).

| Algorithm | tp | fp | tn | fn | Accuracy | Precision | Specificity | Sensitivity | NPV | MCC |
| --- | --- | --- | --- | --- | --- | --- | --- | --- | --- | --- |
| Disease-Specific (All) | 2885 | 1292 | 82101 | 623 | 0.9 | 0.98 | 0.98 | 0.82 | 0.85 | 0.82 |
| Naive (All) | 2813 | 832 | 82561 | 695 | 0.9 | 0.99 | 0.99 | 0.8 | 0.83 | 0.81 |
| Disease-Specific (5) | 171 | 458 | 11332 | 28 | 0.91 | 0.96 | 0.96 | 0.86 | 0.87 | 0.82 |
| Naive (5) | 131 | 111 | 11679 | 68 | 0.82 | 0.99 | 0.99 | 0.66 | 0.74 | 0.69 |
| Disease-Specific (10) | 402 | 643 | 17984 | 71 | 0.91 | 0.96 | 0.97 | 0.85 | 0.87 | 0.82 |
| Naive (10) | 339 | 211 | 18416 | 134 | 0.85 | 0.98 | 0.99 | 0.72 | 0.78 | 0.73 |
| Disease-Specific (25) | 586 | 911 | 28494 | 179 | 0.87 | 0.96 | 0.97 | 0.77 | 0.81 | 0.75 |
| Naive (52) | 432 | 258 | 29147 | 333 | 0.78 | 0.98 | 0.99 | 0.56 | 0.69 | 0.61 |
| Disease-Specific (100) | 1080 | 1178 | 50731 | 316 | 0.88 | 0.97 | 0.98 | 0.77 | 0.81 | 0.77 |
| Naive (100) | 862 | 342 | 51567 | 534 | 0.81 | 0.99 | 0.99 | 0.62 | 0.72 | 0.66 |
| Disease-Specific (250) | 1478 | 1301 | 63683 | 449 | 0.87 | 0.97 | 0.98 | 0.77 | 0.81 | 0.76 |
| Naive (250) | 1257 | 443 | 64541 | 670 | 0.82 | 0.99 | 0.99 | 0.65 | 0.74 | 0.69 |

In the above, *tp*, *fp*, *tn* and *fn* refer to the number of true positive, false positive, true negative and false negatives observed. *Accuracy*, *Precision*, *Specificity*, *Sensitivity*, Negative Predictive Value (*NPV*) and Matthew’s Correlation Coefficient (*MCC*) were calculated using normalized numbers. The number shown in brackets refer to the maximum difference between disease-associated mutations and other germline polymorphisms allowed in the weighting scheme; the higher this number is, the more imbalanced the weighting scheme is.

**Supp. Info 22.** Performance of our Disease-Specific Approach and Naive Weighting Scheme when using Balanced and Imbalanced Weights (Developmental Disease Mutations).

| Algorithm | tp | fp | tn | fn | Accuracy | Precision | Specificity | Sensitivity | NPV | MCC |
| --- | --- | --- | --- | --- | --- | --- | --- | --- | --- | --- |
| Disease-Specific (All) | 621 | 710 | 84997 | 573 | 0.76 | 0.98 | 0.99 | 0.52 | 0.67 | 0.58 |
| Naive (All) | 599 | 272 | 85435 | 595 | 0.75 | 0.99 | 1 | 0.5 | 0.67 | 0.57 |
| Disease-Specific (5) | 167 | 413 | 11340 | 76 | 0.83 | 0.95 | 0.96 | 0.69 | 0.76 | 0.68 |
| Naive (5) | 123 | 59 | 11694 | 120 | 0.75 | 0.99 | 0.99 | 0.51 | 0.67 | 0.57 |
| Disease-Specific (10) | 267 | 520 | 17949 | 121 | 0.83 | 0.96 | 0.97 | 0.69 | 0.76 | 0.69 |
| Naive (10) | 222 | 92 | 18377 | 166 | 0.78 | 0.99 | 1 | 0.57 | 0.7 | 0.63 |
| Disease-Specific (25) | 481 | 738 | 28255 | 225 | 0.83 | 0.96 | 0.97 | 0.68 | 0.75 | 0.69 |
| Naive (52) | 415 | 196 | 28797 | 291 | 0.79 | 0.99 | 0.99 | 0.59 | 0.71 | 0.64 |
| Disease-Specific (100) | 670 | 941 | 50514 | 350 | 0.82 | 0.97 | 0.98 | 0.66 | 0.74 | 0.68 |
| Naive (100) | 599 | 272 | 51183 | 421 | 0.79 | 0.99 | 0.99 | 0.59 | 0.71 | 0.64 |
| Disease-Specific (250) | 670 | 1038 | 64483 | 414 | 0.8 | 0.98 | 0.98 | 0.62 | 0.72 | 0.65 |
| Naive (250) | 599 | 272 | 65249 | 485 | 0.77 | 0.99 | 1 | 0.55 | 0.69 | 0.61 |

In the above, *tp*, *fp*, *tn* and *fn* refer to the number of true positive, false positive, true negative and false negatives observed. *Accuracy*, *Precision*, *Specificity*, *Sensitivity*, Negative Predictive Value (*NPV*) and Matthew’s Correlation Coefficient (*MCC*) were calculated using normalized numbers. The number shown in brackets refer to the maximum difference between disease-associated mutations and other germline polymorphisms allowed in the weighting scheme; the higher this number is, the more imbalanced the weighting scheme is.

**Supp. Info 23.** Performance of our Disease-Specific Approach and Naive Weighting Scheme when using Balanced and Imbalanced Weights (Digestive Disease Mutations).

| Algorithm | tp | fp | tn | fn | Accuracy | Precision | Specificity | Sensitivity | NPV | MCC |
| --- | --- | --- | --- | --- | --- | --- | --- | --- | --- | --- |
| Disease-Specific (All) | 844 | 734 | 84379 | 944 | 0.73 | 0.98 | 0.99 | 0.47 | 0.65 | 0.54 |
| Naive (All) | 808 | 248 | 84865 | 980 | 0.72 | 0.99 | 1 | 0.45 | 0.65 | 0.54 |
| Disease-Specific (5) | 150 | 423 | 11332 | 61 | 0.84 | 0.95 | 0.96 | 0.71 | 0.77 | 0.7 |
| Naive (5) | 93 | 55 | 11700 | 118 | 0.72 | 0.99 | 1 | 0.44 | 0.64 | 0.52 |
| Disease-Specific (10) | 228 | 572 | 18010 | 121 | 0.81 | 0.96 | 0.97 | 0.65 | 0.74 | 0.66 |
| Naive (10) | 116 | 63 | 18519 | 233 | 0.66 | 0.99 | 1 | 0.33 | 0.6 | 0.44 |
| Disease-Specific (25) | 525 | 771 | 28171 | 190 | 0.85 | 0.96 | 0.97 | 0.73 | 0.79 | 0.73 |
| Naive (52) | 406 | 146 | 28796 | 309 | 0.78 | 0.99 | 0.99 | 0.57 | 0.7 | 0.62 |
| Disease-Specific (100) | 831 | 991 | 50660 | 347 | 0.84 | 0.97 | 0.98 | 0.71 | 0.77 | 0.71 |
| Naive (100) | 700 | 221 | 51430 | 478 | 0.79 | 0.99 | 1 | 0.59 | 0.71 | 0.64 |
| Disease-Specific (250) | 949 | 1127 | 64583 | 499 | 0.82 | 0.97 | 0.98 | 0.66 | 0.74 | 0.68 |
| Naive (250) | 808 | 248 | 65462 | 640 | 0.78 | 0.99 | 1 | 0.56 | 0.69 | 0.62 |

In the above, *tp*, *fp*, *tn* and *fn* refer to the number of true positive, false positive, true negative and false negatives observed. *Accuracy*, *Precision*, *Specificity*, *Sensitivity*, Negative Predictive Value (*NPV*) and Matthew’s Correlation Coefficient (*MCC*) were calculated using normalized numbers. The number shown in brackets refer to the maximum difference between disease-associated mutations and other germline polymorphisms allowed in the weighting scheme; the higher this number is, the more imbalanced the weighting scheme is.

**Supp. Info 24.** Performance of our Disease-Specific Approach and Naive Weighting Scheme when using Balanced and Imbalanced Weights (Ear, Nose and Throat Disease Mutations).

| Algorithm | tp | fp | tn | fn | Accuracy | Precision | Specificity | Sensitivity | NPV | MCC |
| --- | --- | --- | --- | --- | --- | --- | --- | --- | --- | --- |
| Disease-Specific (All) | 192 | 199 | 85762 | 748 | 0.6 | 0.99 | 1 | 0.2 | 0.56 | 0.33 |
| Naive (All) | 281 | 65 | 85896 | 659 | 0.65 | 1 | 1 | 0.3 | 0.59 | 0.42 |
| Disease-Specific (5) | 94 | 353 | 11285 | 36 | 0.85 | 0.96 | 0.97 | 0.72 | 0.78 | 0.71 |
| Naive (5) | 73 | 27 | 11611 | 57 | 0.78 | 1 | 1 | 0.56 | 0.69 | 0.62 |
| Disease-Specific (10) | 126 | 437 | 17927 | 69 | 0.81 | 0.96 | 0.98 | 0.65 | 0.73 | 0.66 |
| Naive (10) | 105 | 32 | 18332 | 90 | 0.77 | 1 | 1 | 0.54 | 0.68 | 0.6 |
| Disease-Specific (25) | 149 | 539 | 28273 | 114 | 0.77 | 0.97 | 0.98 | 0.57 | 0.69 | 0.6 |
| Naive (52) | 123 | 36 | 28776 | 140 | 0.73 | 1 | 1 | 0.47 | 0.65 | 0.55 |
| Disease-Specific (100) | 322 | 662 | 51229 | 321 | 0.74 | 0.98 | 0.99 | 0.5 | 0.66 | 0.56 |
| Naive (100) | 281 | 65 | 51826 | 362 | 0.72 | 1 | 1 | 0.44 | 0.64 | 0.53 |
| Disease-Specific (250) | 329 | 771 | 65507 | 526 | 0.69 | 0.97 | 0.99 | 0.38 | 0.62 | 0.47 |
| Naive (250) | 281 | 65 | 66213 | 574 | 0.66 | 1 | 1 | 0.33 | 0.6 | 0.44 |

In the above, *tp*, *fp*, *tn* and *fn* refer to the number of true positive, false positive, true negative and false negatives observed. *Accuracy*, *Precision*, *Specificity*, *Sensitivity*, Negative Predictive Value (*NPV*) and Matthew’s Correlation Coefficient (*MCC*) were calculated using normalized numbers. The number shown in brackets refer to the maximum difference between disease-associated mutations and other germline polymorphisms allowed in the weighting scheme; the higher this number is, the more imbalanced the weighting scheme is.

**Supp. Info 25.** Performance of our Disease-Specific Approach and Naive Weighting Scheme when using Balanced and Imbalanced Weights (Endocrine Disease Mutations).

| Algorithm | tp | fp | tn | fn | Accuracy | Precision | Specificity | Sensitivity | NPV | MCC |
| --- | --- | --- | --- | --- | --- | --- | --- | --- | --- | --- |
| Disease-Specific (All) | 2392 | 1015 | 81973 | 1521 | 0.8 | 0.98 | 0.99 | 0.61 | 0.72 | 0.65 |
| Naive (All) | 2388 | 610 | 82378 | 1525 | 0.8 | 0.99 | 0.99 | 0.61 | 0.72 | 0.65 |
| Disease-Specific (5) | 241 | 457 | 11334 | 59 | 0.88 | 0.95 | 0.96 | 0.8 | 0.83 | 0.77 |
| Naive (5) | 194 | 112 | 11679 | 106 | 0.82 | 0.99 | 0.99 | 0.65 | 0.74 | 0.68 |
| Disease-Specific (10) | 348 | 599 | 17925 | 107 | 0.87 | 0.96 | 0.97 | 0.76 | 0.8 | 0.75 |
| Naive (10) | 287 | 174 | 18350 | 168 | 0.81 | 0.99 | 0.99 | 0.63 | 0.73 | 0.67 |
| Disease-Specific (25) | 777 | 828 | 27993 | 168 | 0.9 | 0.97 | 0.97 | 0.82 | 0.85 | 0.8 |
| Naive (52) | 706 | 302 | 28519 | 239 | 0.87 | 0.99 | 0.99 | 0.75 | 0.8 | 0.76 |
| Disease-Specific (100) | 1297 | 1156 | 50143 | 525 | 0.84 | 0.97 | 0.98 | 0.71 | 0.77 | 0.72 |
| Naive (100) | 1193 | 498 | 50801 | 629 | 0.82 | 0.99 | 0.99 | 0.65 | 0.74 | 0.68 |
| Disease-Specific (250) | 2517 | 1457 | 63000 | 764 | 0.87 | 0.97 | 0.98 | 0.77 | 0.81 | 0.76 |
| Naive (250) | 2382 | 608 | 63849 | 899 | 0.86 | 0.99 | 0.99 | 0.73 | 0.78 | 0.74 |

In the above, *tp*, *fp*, *tn* and *fn* refer to the number of true positive, false positive, true negative and false negatives observed. *Accuracy*, *Precision*, *Specificity*, *Sensitivity*, Negative Predictive Value (*NPV*) and Matthew’s Correlation Coefficient (*MCC*) were calculated using normalized numbers. The number shown in brackets refer to the maximum difference between disease-associated mutations and other germline polymorphisms allowed in the weighting scheme; the higher this number is, the more imbalanced the weighting scheme is.

**Supp. Info 26.** Performance of our Disease-Specific Approach and Naive Weighting Scheme when using Balanced and Imbalanced Weights (Eye Disease Mutations).

| Algorithm | tp | fp | tn | fn | Accuracy | Precision | Specificity | Sensitivity | NPV | MCC |
| --- | --- | --- | --- | --- | --- | --- | --- | --- | --- | --- |
| Disease-Specific (All) | 1433 | 1127 | 82744 | 1597 | 0.73 | 0.97 | 0.99 | 0.47 | 0.65 | 0.54 |
| Naive (All) | 1338 | 488 | 83383 | 1692 | 0.72 | 0.99 | 0.99 | 0.44 | 0.64 | 0.52 |
| Disease-Specific (5) | 337 | 585 | 11390 | 131 | 0.84 | 0.94 | 0.95 | 0.72 | 0.77 | 0.69 |
| Naive (5) | 236 | 146 | 11829 | 232 | 0.75 | 0.98 | 0.99 | 0.5 | 0.67 | 0.56 |
| Disease-Specific (10) | 525 | 785 | 17962 | 182 | 0.85 | 0.95 | 0.96 | 0.74 | 0.79 | 0.72 |
| Naive (10) | 417 | 250 | 18497 | 290 | 0.79 | 0.98 | 0.99 | 0.59 | 0.71 | 0.63 |
| Disease-Specific (25) | 905 | 1094 | 28133 | 336 | 0.85 | 0.95 | 0.96 | 0.73 | 0.78 | 0.71 |
| Naive (52) | 737 | 371 | 28856 | 504 | 0.79 | 0.98 | 0.99 | 0.59 | 0.71 | 0.63 |
| Disease-Specific (100) | 1409 | 1392 | 49912 | 639 | 0.83 | 0.96 | 0.97 | 0.69 | 0.76 | 0.69 |
| Naive (100) | 1168 | 461 | 50843 | 880 | 0.78 | 0.98 | 0.99 | 0.57 | 0.7 | 0.62 |
| Disease-Specific (250) | 1608 | 1589 | 62911 | 850 | 0.81 | 0.96 | 0.98 | 0.65 | 0.74 | 0.66 |
| Naive (250) | 1338 | 488 | 64012 | 1120 | 0.77 | 0.99 | 0.99 | 0.54 | 0.69 | 0.6 |

In the above, *tp*, *fp*, *tn* and *fn* refer to the number of true positive, false positive, true negative and false negatives observed. *Accuracy*, *Precision*, *Specificity*, *Sensitivity*, Negative Predictive Value (*NPV*) and Matthew’s Correlation Coefficient (*MCC*) were calculated using normalized numbers. The number shown in brackets refer to the maximum difference between disease-associated mutations and other germline polymorphisms allowed in the weighting scheme; the higher this number is, the more imbalanced the weighting scheme is.

**Supp. Info 27.** Performance of our Disease-Specific Approach and Naive Weighting Scheme when using Balanced and Imbalanced Weights (Genitourinary Disease Mutations).

| Algorithm | tp | fp | tn | fn | Accuracy | Precision | Specificity | Sensitivity | NPV | MCC |
| --- | --- | --- | --- | --- | --- | --- | --- | --- | --- | --- |
| Disease-Specific (All) | 1601 | 1327 | 82544 | 1429 | 0.76 | 0.97 | 0.98 | 0.53 | 0.68 | 0.58 |
| Naive (All) | 1410 | 377 | 83494 | 1620 | 0.73 | 0.99 | 1 | 0.47 | 0.65 | 0.54 |
| Disease-Specific (5) | 240 | 481 | 11329 | 60 | 0.88 | 0.95 | 0.96 | 0.8 | 0.83 | 0.77 |
| Naive (5) | 193 | 124 | 11686 | 107 | 0.82 | 0.98 | 0.99 | 0.64 | 0.74 | 0.67 |
| Disease-Specific (10) | 389 | 654 | 17905 | 138 | 0.85 | 0.95 | 0.96 | 0.74 | 0.79 | 0.72 |
| Naive (10) | 311 | 195 | 18364 | 216 | 0.79 | 0.98 | 0.99 | 0.59 | 0.71 | 0.63 |
| Disease-Specific (25) | 677 | 874 | 28188 | 248 | 0.85 | 0.96 | 0.97 | 0.73 | 0.78 | 0.72 |
| Naive (52) | 584 | 306 | 28756 | 341 | 0.81 | 0.98 | 0.99 | 0.63 | 0.73 | 0.66 |
| Disease-Specific (100) | 1107 | 1074 | 50054 | 672 | 0.8 | 0.97 | 0.98 | 0.62 | 0.72 | 0.64 |
| Naive (100) | 954 | 342 | 50786 | 825 | 0.76 | 0.99 | 0.99 | 0.54 | 0.68 | 0.6 |
| Disease-Specific (250) | 1423 | 1226 | 63419 | 849 | 0.8 | 0.97 | 0.98 | 0.63 | 0.72 | 0.65 |
| Naive (250) | 1256 | 376 | 64269 | 1016 | 0.77 | 0.99 | 0.99 | 0.55 | 0.69 | 0.61 |

In the above, *tp*, *fp*, *tn* and *fn* refer to the number of true positive, false positive, true negative and false negatives observed. *Accuracy*, *Precision*, *Specificity*, *Sensitivity*, Negative Predictive Value (*NPV*) and Matthew’s Correlation Coefficient (*MCC*) were calculated using normalized numbers. The number shown in brackets refer to the maximum difference between disease-associated mutations and other germline polymorphisms allowed in the weighting scheme; the higher this number is, the more imbalanced the weighting scheme is.

**Supp. Info 28.** Performance of our Disease-Specific Approach and Naive Weighting Scheme when using Balanced and Imbalanced Weights (Heart Disease Mutations).

| Algorithm | tp | fp | tn | fn | Accuracy | Precision | Specificity | Sensitivity | NPV | MCC |
| --- | --- | --- | --- | --- | --- | --- | --- | --- | --- | --- |
| Disease-Specific (All) | 2536 | 2885 | 80274 | 1206 | 0.82 | 0.95 | 0.97 | 0.68 | 0.75 | 0.67 |
| Naive (All) | 1748 | 928 | 82231 | 1994 | 0.73 | 0.98 | 0.99 | 0.47 | 0.65 | 0.53 |
| Disease-Specific (5) | 355 | 588 | 11360 | 116 | 0.85 | 0.94 | 0.95 | 0.75 | 0.79 | 0.72 |
| Naive (5) | 240 | 161 | 11787 | 231 | 0.75 | 0.97 | 0.99 | 0.51 | 0.67 | 0.56 |
| Disease-Specific (10) | 521 | 752 | 17895 | 180 | 0.85 | 0.95 | 0.96 | 0.74 | 0.79 | 0.72 |
| Naive (10) | 357 | 200 | 18447 | 344 | 0.75 | 0.98 | 0.99 | 0.51 | 0.67 | 0.57 |
| Disease-Specific (25) | 1211 | 1431 | 28416 | 423 | 0.85 | 0.94 | 0.95 | 0.74 | 0.79 | 0.71 |
| Naive (52) | 778 | 446 | 29401 | 856 | 0.73 | 0.97 | 0.99 | 0.48 | 0.65 | 0.54 |
| Disease-Specific (100) | 2068 | 2093 | 49707 | 722 | 0.85 | 0.95 | 0.96 | 0.74 | 0.79 | 0.72 |
| Naive (100) | 1572 | 875 | 50925 | 1218 | 0.77 | 0.97 | 0.98 | 0.56 | 0.69 | 0.6 |
| Disease-Specific (250) | 2286 | 2295 | 63736 | 1132 | 0.82 | 0.95 | 0.97 | 0.67 | 0.74 | 0.66 |
| Naive (250) | 1748 | 928 | 65103 | 1670 | 0.75 | 0.97 | 0.99 | 0.51 | 0.67 | 0.57 |

In the above, *tp*, *fp*, *tn* and *fn* refer to the number of true positive, false positive, true negative and false negatives observed. *Accuracy*, *Precision*, *Specificity*, *Sensitivity*, Negative Predictive Value (*NPV*) and Matthew’s Correlation Coefficient (*MCC*) were calculated using normalized numbers. The number shown in brackets refer to the maximum difference between disease-associated mutations and other germline polymorphisms allowed in the weighting scheme; the higher this number is, the more imbalanced the weighting scheme is.

**Supp. Info 29.** Performance of our Disease-Specific Approach and Naive Weighting Scheme when using Balanced and Imbalanced Weights (Immune Disease Mutations).

| Algorithm | tp | fp | tn | fn | Accuracy | Precision | Specificity | Sensitivity | NPV | MCC |
| --- | --- | --- | --- | --- | --- | --- | --- | --- | --- | --- |
| Disease-Specific (All) | 590 | 385 | 85223 | 703 | 0.73 | 0.99 | 1 | 0.46 | 0.65 | 0.54 |
| Naive (All) | 677 | 160 | 85448 | 616 | 0.76 | 1 | 1 | 0.52 | 0.68 | 0.59 |
| Disease-Specific (5) | 160 | 426 | 11374 | 48 | 0.87 | 0.96 | 0.96 | 0.77 | 0.81 | 0.75 |
| Naive (5) | 87 | 55 | 11745 | 121 | 0.71 | 0.99 | 1 | 0.42 | 0.63 | 0.51 |
| Disease-Specific (10) | 220 | 510 | 17992 | 92 | 0.84 | 0.96 | 0.97 | 0.71 | 0.77 | 0.7 |
| Naive (10) | 134 | 64 | 18438 | 178 | 0.71 | 0.99 | 1 | 0.43 | 0.64 | 0.52 |
| Disease-Specific (25) | 395 | 654 | 28218 | 149 | 0.85 | 0.97 | 0.98 | 0.73 | 0.78 | 0.73 |
| Naive (52) | 295 | 100 | 28772 | 249 | 0.77 | 0.99 | 1 | 0.54 | 0.69 | 0.6 |
| Disease-Specific (100) | 759 | 850 | 50386 | 263 | 0.86 | 0.98 | 0.98 | 0.74 | 0.79 | 0.75 |
| Naive (100) | 652 | 158 | 51078 | 370 | 0.82 | 1 | 1 | 0.64 | 0.73 | 0.68 |
| Disease-Specific (250) | 815 | 1037 | 64514 | 363 | 0.84 | 0.98 | 0.98 | 0.69 | 0.76 | 0.71 |
| Naive (250) | 677 | 160 | 65391 | 501 | 0.79 | 1 | 1 | 0.57 | 0.7 | 0.63 |

In the above, *tp*, *fp*, *tn* and *fn* refer to the number of true positive, false positive, true negative and false negatives observed. *Accuracy*, *Precision*, *Specificity*, *Sensitivity*, Negative Predictive Value (*NPV*) and Matthew’s Correlation Coefficient (*MCC*) were calculated using normalized numbers. The number shown in brackets refer to the maximum difference between disease-associated mutations and other germline polymorphisms allowed in the weighting scheme; the higher this number is, the more imbalanced the weighting scheme is.

**Supp. Info 30.** Performance of our Disease-Specific Approach and Naive Weighting Scheme when using Balanced and Imbalanced Weights (Metabolic Disease Mutations).

| Algorithm | tp | fp | tn | fn | Accuracy | Precision | Specificity | Sensitivity | NPV | MCC |
| --- | --- | --- | --- | --- | --- | --- | --- | --- | --- | --- |
| Disease-Specific (All) | 10767 | 3209 | 69976 | 2949 | 0.87 | 0.95 | 0.96 | 0.78 | 0.82 | 0.75 |
| Naive (All) | 10716 | 2731 | 70454 | 3000 | 0.87 | 0.95 | 0.96 | 0.78 | 0.81 | 0.76 |
| Disease-Specific (5) | 890 | 844 | 11310 | 186 | 0.88 | 0.92 | 0.93 | 0.83 | 0.84 | 0.76 |
| Naive (5) | 704 | 386 | 11768 | 372 | 0.81 | 0.95 | 0.97 | 0.65 | 0.74 | 0.66 |
| Disease-Specific (10) | 1559 | 1230 | 17585 | 307 | 0.89 | 0.93 | 0.93 | 0.84 | 0.85 | 0.77 |
| Naive (10) | 1339 | 659 | 18156 | 527 | 0.84 | 0.95 | 0.96 | 0.72 | 0.77 | 0.7 |
| Disease-Specific (25) | 2984 | 1769 | 26618 | 595 | 0.89 | 0.93 | 0.94 | 0.83 | 0.85 | 0.78 |
| Naive (52) | 2668 | 1032 | 27355 | 911 | 0.85 | 0.95 | 0.96 | 0.75 | 0.79 | 0.73 |
| Disease-Specific (100) | 7711 | 3361 | 44706 | 1340 | 0.89 | 0.92 | 0.93 | 0.85 | 0.86 | 0.78 |
| Naive (100) | 7249 | 2269 | 45798 | 1802 | 0.88 | 0.94 | 0.95 | 0.8 | 0.83 | 0.76 |
| Disease-Specific (250) | 10601 | 4218 | 54020 | 1761 | 0.89 | 0.92 | 0.93 | 0.86 | 0.87 | 0.79 |
| Naive (250) | 9878 | 2586 | 55652 | 2484 | 0.88 | 0.95 | 0.96 | 0.8 | 0.83 | 0.76 |

In the above, *tp*, *fp*, *tn* and *fn* refer to the number of true positive, false positive, true negative and false negatives observed. *Accuracy*, *Precision*, *Specificity*, *Sensitivity*, Negative Predictive Value (*NPV*) and Matthew’s Correlation Coefficient (*MCC*) were calculated using normalized numbers. The number shown in brackets refer to the maximum difference between disease-associated mutations and other germline polymorphisms allowed in the weighting scheme; the higher this number is, the more imbalanced the weighting scheme is.

**Supp. Info 31.** Performance of our Disease-Specific Approach and Naive Weighting Scheme when using Balanced and Imbalanced Weights (Musculoskeletal Disease Mutations).

| Algorithm | tp | fp | tn | fn | Accuracy | Precision | Specificity | Sensitivity | NPV | MCC |
| --- | --- | --- | --- | --- | --- | --- | --- | --- | --- | --- |
| Disease-Specific (All) | 4120 | 3123 | 77675 | 1983 | 0.82 | 0.95 | 0.96 | 0.68 | 0.75 | 0.66 |
| Naive (All) | 3697 | 1868 | 78930 | 2406 | 0.79 | 0.96 | 0.98 | 0.61 | 0.71 | 0.63 |
| Disease-Specific (5) | 557 | 673 | 11410 | 116 | 0.89 | 0.94 | 0.94 | 0.83 | 0.85 | 0.78 |
| Naive (5) | 370 | 213 | 11870 | 303 | 0.77 | 0.97 | 0.98 | 0.55 | 0.69 | 0.59 |
| Disease-Specific (10) | 951 | 1019 | 18021 | 288 | 0.86 | 0.93 | 0.95 | 0.77 | 0.8 | 0.73 |
| Naive (10) | 640 | 335 | 18705 | 599 | 0.75 | 0.97 | 0.98 | 0.52 | 0.67 | 0.56 |
| Disease-Specific (25) | 1417 | 1369 | 27914 | 449 | 0.86 | 0.94 | 0.95 | 0.76 | 0.8 | 0.73 |
| Naive (52) | 1081 | 560 | 28723 | 785 | 0.78 | 0.97 | 0.98 | 0.58 | 0.7 | 0.61 |
| Disease-Specific (100) | 2911 | 2244 | 48396 | 975 | 0.85 | 0.94 | 0.96 | 0.75 | 0.79 | 0.72 |
| Naive (100) | 2498 | 1202 | 49438 | 1388 | 0.81 | 0.96 | 0.98 | 0.64 | 0.73 | 0.66 |
| Disease-Specific (250) | 3508 | 2910 | 61583 | 1489 | 0.83 | 0.94 | 0.95 | 0.7 | 0.76 | 0.68 |
| Naive (250) | 3004 | 1483 | 63010 | 1993 | 0.79 | 0.96 | 0.98 | 0.6 | 0.71 | 0.62 |

In the above, *tp*, *fp*, *tn* and *fn* refer to the number of true positive, false positive, true negative and false negatives observed. *Accuracy*, *Precision*, *Specificity*, *Sensitivity*, Negative Predictive Value (*NPV*) and Matthew’s Correlation Coefficient (*MCC*) were calculated using normalized numbers. The number shown in brackets refer to the maximum difference between disease-associated mutations and other germline polymorphisms allowed in the weighting scheme; the higher this number is, the more imbalanced the weighting scheme is.

**Supp. Info 32.** Performance of our Disease-Specific Approach and Naive Weighting Scheme when using Balanced and Imbalanced Weights (Nervous System Disease Mutations).

| Algorithm | tp | fp | tn | fn | Accuracy | Precision | Specificity | Sensitivity | NPV | MCC |
| --- | --- | --- | --- | --- | --- | --- | --- | --- | --- | --- |
| Disease-Specific (All) | 5560 | 2419 | 75931 | 2991 | 0.81 | 0.95 | 0.97 | 0.65 | 0.73 | 0.65 |
| Naive (All) | 5230 | 1751 | 76599 | 3321 | 0.79 | 0.96 | 0.98 | 0.61 | 0.72 | 0.63 |
| Disease-Specific (5) | 878 | 904 | 11410 | 227 | 0.86 | 0.92 | 0.93 | 0.79 | 0.82 | 0.73 |
| Naive (5) | 562 | 295 | 12019 | 543 | 0.74 | 0.96 | 0.98 | 0.51 | 0.67 | 0.55 |
| Disease-Specific (10) | 1316 | 1216 | 17845 | 376 | 0.86 | 0.92 | 0.94 | 0.78 | 0.81 | 0.72 |
| Naive (10) | 966 | 525 | 18536 | 726 | 0.77 | 0.95 | 0.97 | 0.57 | 0.69 | 0.59 |
| Disease-Specific (25) | 2430 | 1824 | 27492 | 678 | 0.86 | 0.93 | 0.94 | 0.78 | 0.81 | 0.73 |
| Naive (52) | 2019 | 984 | 28332 | 1089 | 0.81 | 0.95 | 0.97 | 0.65 | 0.73 | 0.65 |
| Disease-Specific (100) | 4564 | 2732 | 46541 | 1370 | 0.86 | 0.93 | 0.94 | 0.77 | 0.8 | 0.72 |
| Naive (100) | 4024 | 1600 | 47673 | 1910 | 0.82 | 0.95 | 0.97 | 0.68 | 0.75 | 0.67 |
| Disease-Specific (250) | 5391 | 2868 | 58016 | 1701 | 0.86 | 0.94 | 0.95 | 0.76 | 0.8 | 0.73 |
| Naive (250) | 4841 | 1704 | 59180 | 2251 | 0.83 | 0.96 | 0.97 | 0.68 | 0.75 | 0.68 |

In the above, *tp*, *fp*, *tn* and *fn* refer to the number of true positive, false positive, true negative and false negatives observed. *Accuracy*, *Precision*, *Specificity*, *Sensitivity*, Negative Predictive Value (*NPV*) and Matthew’s Correlation Coefficient (*MCC*) were calculated using normalized numbers. The number shown in brackets refer to the maximum difference between disease-associated mutations and other germline polymorphisms allowed in the weighting scheme; the higher this number is, the more imbalanced the weighting scheme is.

**Supp. Info 33.** Performance of our Disease-Specific Approach and Naive Weighting Scheme when using Balanced and Imbalanced Weights (Psychiatric Disease Mutations).

| Algorithm | tp | fp | tn | fn | Accuracy | Precision | Specificity | Sensitivity | NPV | MCC |
| --- | --- | --- | --- | --- | --- | --- | --- | --- | --- | --- |
| Disease-Specific (All) | 379 | 165 | 85990 | 367 | 0.75 | 1 | 1 | 0.51 | 0.67 | 0.58 |
| Naive (All) | 483 | 108 | 86047 | 263 | 0.82 | 1 | 1 | 0.65 | 0.74 | 0.69 |
| Disease-Specific (5) | 104 | 355 | 11270 | 34 | 0.86 | 0.96 | 0.97 | 0.75 | 0.8 | 0.74 |
| Naive (5) | 79 | 25 | 11600 | 59 | 0.79 | 1 | 1 | 0.57 | 0.7 | 0.63 |
| Disease-Specific (10) | 138 | 440 | 17866 | 58 | 0.84 | 0.97 | 0.98 | 0.7 | 0.77 | 0.71 |
| Naive (10) | 110 | 32 | 18274 | 86 | 0.78 | 1 | 1 | 0.56 | 0.69 | 0.62 |
| Disease-Specific (25) | 188 | 538 | 28278 | 114 | 0.8 | 0.97 | 0.98 | 0.62 | 0.72 | 0.65 |
| Naive (52) | 157 | 39 | 28777 | 145 | 0.76 | 1 | 1 | 0.52 | 0.68 | 0.59 |
| Disease-Specific (100) | 325 | 683 | 51087 | 184 | 0.81 | 0.98 | 0.99 | 0.64 | 0.73 | 0.67 |
| Naive (100) | 293 | 96 | 51674 | 216 | 0.79 | 1 | 1 | 0.58 | 0.7 | 0.63 |
| Disease-Specific (250) | 515 | 792 | 64967 | 211 | 0.85 | 0.98 | 0.99 | 0.71 | 0.77 | 0.73 |
| Naive (250) | 483 | 108 | 65651 | 243 | 0.83 | 1 | 1 | 0.67 | 0.75 | 0.7 |

In the above, *tp*, *fp*, *tn* and *fn* refer to the number of true positive, false positive, true negative and false negatives observed. *Accuracy*, *Precision*, *Specificity*, *Sensitivity*, Negative Predictive Value (*NPV*) and Matthew’s Correlation Coefficient (*MCC*) were calculated using normalized numbers. The number shown in brackets refer to the maximum difference between disease-associated mutations and other germline polymorphisms allowed in the weighting scheme; the higher this number is, the more imbalanced the weighting scheme is.

**Supp. Info 34.** Performance of our Disease-Specific Approach and Naive Weighting Scheme when using Balanced and Imbalanced Weights (Reproductive Disease Mutations).

| Algorithm | tp | fp | tn | fn | Accuracy | Precision | Specificity | Sensitivity | NPV | MCC |
| --- | --- | --- | --- | --- | --- | --- | --- | --- | --- | --- |
| Disease-Specific (All) | 131 | 36 | 85982 | 752 | 0.57 | 1 | 1 | 0.15 | 0.54 | 0.28 |
| Naive (All) | 233 | 78 | 85940 | 650 | 0.63 | 1 | 1 | 0.26 | 0.58 | 0.39 |
| Disease-Specific (5) | 111 | 393 | 11301 | 28 | 0.88 | 0.96 | 0.97 | 0.8 | 0.83 | 0.78 |
| Naive (5) | 94 | 62 | 11632 | 45 | 0.84 | 0.99 | 0.99 | 0.68 | 0.75 | 0.71 |
| Disease-Specific (10) | 133 | 484 | 17915 | 49 | 0.85 | 0.97 | 0.97 | 0.73 | 0.78 | 0.73 |
| Naive (10) | 109 | 76 | 18323 | 73 | 0.8 | 0.99 | 1 | 0.6 | 0.71 | 0.65 |
| Disease-Specific (25) | 157 | 590 | 28359 | 106 | 0.79 | 0.97 | 0.98 | 0.6 | 0.71 | 0.62 |
| Naive (52) | 125 | 76 | 28873 | 138 | 0.74 | 0.99 | 1 | 0.48 | 0.66 | 0.55 |
| Disease-Specific (100) | 172 | 720 | 51152 | 294 | 0.68 | 0.96 | 0.99 | 0.37 | 0.61 | 0.45 |
| Naive (100) | 125 | 76 | 51796 | 341 | 0.63 | 0.99 | 1 | 0.27 | 0.58 | 0.39 |
| Disease-Specific (250) | 280 | 819 | 65287 | 364 | 0.71 | 0.97 | 0.99 | 0.43 | 0.64 | 0.51 |
| Naive (250) | 233 | 78 | 66028 | 411 | 0.68 | 1 | 1 | 0.36 | 0.61 | 0.47 |

In the above, *tp*, *fp*, *tn* and *fn* refer to the number of true positive, false positive, true negative and false negatives observed. *Accuracy*, *Precision*, *Specificity*, *Sensitivity*, Negative Predictive Value (*NPV*) and Matthew’s Correlation Coefficient (*MCC*) were calculated using normalized numbers. The number shown in brackets refer to the maximum difference between disease-associated mutations and other germline polymorphisms allowed in the weighting scheme; the higher this number is, the more imbalanced the weighting scheme is.

**Supp. Info 35.** Performance of our Disease-Specific Approach and Naive Weighting Scheme when using Balanced and Imbalanced Weights (Respiratory Disease Mutations).

| Algorithm | tp | fp | tn | fn | Accuracy | Precision | Specificity | Sensitivity | NPV | MCC |
| --- | --- | --- | --- | --- | --- | --- | --- | --- | --- | --- |
| Disease-Specific (All) | 478 | 1604 | 84522 | 297 | 0.8 | 0.97 | 0.98 | 0.62 | 0.72 | 0.64 |
| Naive (All) | 162 | 71 | 86055 | 613 | 0.6 | 1 | 1 | 0.21 | 0.56 | 0.34 |
| Disease-Specific (5) | 36 | 342 | 11319 | 19 | 0.81 | 0.96 | 0.97 | 0.65 | 0.74 | 0.66 |
| Naive (5) | 25 | 12 | 11649 | 30 | 0.73 | 1 | 1 | 0.45 | 0.65 | 0.54 |
| Disease-Specific (10) | 49 | 422 | 17941 | 38 | 0.77 | 0.96 | 0.98 | 0.56 | 0.69 | 0.59 |
| Naive (10) | 32 | 12 | 18351 | 55 | 0.68 | 1 | 1 | 0.37 | 0.61 | 0.47 |
| Disease-Specific (25) | 117 | 546 | 28405 | 61 | 0.82 | 0.97 | 0.98 | 0.66 | 0.74 | 0.67 |
| Naive (52) | 94 | 33 | 28918 | 84 | 0.76 | 1 | 1 | 0.53 | 0.68 | 0.6 |
| Disease-Specific (100) | 200 | 695 | 51476 | 265 | 0.71 | 0.97 | 0.99 | 0.43 | 0.63 | 0.5 |
| Naive (100) | 162 | 71 | 52100 | 303 | 0.67 | 1 | 1 | 0.35 | 0.61 | 0.46 |
| Disease-Specific (250) | 224 | 813 | 66049 | 443 | 0.66 | 0.97 | 0.99 | 0.34 | 0.6 | 0.43 |
| Naive (250) | 162 | 71 | 66791 | 505 | 0.62 | 1 | 1 | 0.24 | 0.57 | 0.37 |

In the above, *tp*, *fp*, *tn* and *fn* refer to the number of true positive, false positive, true negative and false negatives observed. *Accuracy*, *Precision*, *Specificity*, *Sensitivity*, Negative Predictive Value (*NPV*) and Matthew’s Correlation Coefficient (*MCC*) were calculated using normalized numbers. The number shown in brackets refer to the maximum difference between disease-associated mutations and other germline polymorphisms allowed in the weighting scheme; the higher this number is, the more imbalanced the weighting scheme is.

**Supp. Info 36.** Performance of our Disease-Specific Approach and Naive Weighting Scheme when using Balanced and Imbalanced Weights (Skin Disease Mutations).

| Algorithm | tp | fp | tn | fn | Accuracy | Precision | Specificity | Sensitivity | NPV | MCC |
| --- | --- | --- | --- | --- | --- | --- | --- | --- | --- | --- |
| Disease-Specific (All) | 1230 | 537 | 83181 | 1953 | 0.69 | 0.98 | 0.99 | 0.39 | 0.62 | 0.48 |
| Naive (All) | 1344 | 397 | 83321 | 1839 | 0.71 | 0.99 | 1 | 0.42 | 0.63 | 0.51 |
| Disease-Specific (5) | 259 | 494 | 11396 | 98 | 0.84 | 0.95 | 0.96 | 0.73 | 0.78 | 0.7 |
| Naive (5) | 146 | 72 | 11818 | 211 | 0.7 | 0.99 | 0.99 | 0.41 | 0.63 | 0.5 |
| Disease-Specific (10) | 480 | 715 | 18079 | 193 | 0.84 | 0.95 | 0.96 | 0.71 | 0.77 | 0.7 |
| Naive (10) | 316 | 169 | 18625 | 357 | 0.73 | 0.98 | 0.99 | 0.47 | 0.65 | 0.54 |
| Disease-Specific (25) | 783 | 962 | 28388 | 324 | 0.84 | 0.96 | 0.97 | 0.71 | 0.77 | 0.7 |
| Naive (52) | 582 | 300 | 29050 | 525 | 0.76 | 0.98 | 0.99 | 0.53 | 0.68 | 0.58 |
| Disease-Specific (100) | 1096 | 1146 | 50052 | 574 | 0.82 | 0.97 | 0.98 | 0.66 | 0.74 | 0.67 |
| Naive (100) | 878 | 341 | 50857 | 792 | 0.76 | 0.99 | 0.99 | 0.53 | 0.68 | 0.59 |
| Disease-Specific (250) | 1434 | 1339 | 63781 | 866 | 0.8 | 0.97 | 0.98 | 0.62 | 0.72 | 0.65 |
| Naive (250) | 1190 | 396 | 64724 | 1110 | 0.76 | 0.99 | 0.99 | 0.52 | 0.67 | 0.58 |

In the above, *tp*, *fp*, *tn* and *fn* refer to the number of true positive, false positive, true negative and false negatives observed. *Accuracy*, *Precision*, *Specificity*, *Sensitivity*, Negative Predictive Value (*NPV*) and Matthew’s Correlation Coefficient (*MCC*) were calculated using normalized numbers. The number shown in brackets refer to the maximum difference between disease-associated mutations and other germline polymorphisms allowed in the weighting scheme; the higher this number is, the more imbalanced the weighting scheme is.
